# Neighborhood environment associations with cognitive function and structural brain measures in older African Americans

**DOI:** 10.1186/s12916-024-03845-7

**Published:** 2025-01-13

**Authors:** Dima L. Chaar, Le Tu, Kari Moore, Jiacong Du, Lauren A Opsasnick, Scott M Ratliff, Thomas H Mosley, Sharon L. R. Kardia, Wei Zhao, Xiang Zhou, Ana V Diez Roux, Fazlay S Faruque, Kenneth R Butler, Jennifer A Smith

**Affiliations:** 1 Department of Epidemiology, School of Public Health, University of Michigan, Ann Arbor, MI USA; 2 Department of Preventive Medicine, John D. Bower School of Population Health, University of Mississippi Medical Center, Jackson, USA; 3 Urban Health Collaborative, Drexel Dornsife School of Public Health, Philadelphia, PA USA; 4 Department of Biostatistics, School of Public Health, University of Michigan, Ann Arbor, MI USA; 5 Memory Impairment and Neurodegenerative Dementia (MIND) Center, University of Mississippi Medical Center, Jackson, MS USA; 6 Survey Research Center, Institute for Social Research, University of Michigan, Ann Arbor, MI USA

**Keywords:** Neighborhood environment, Food environment, Cognitive health, Healthy aging, Cognitive function, White matter hyperintensity, Fast food, Alcohol, Built environment, Social environment

## Abstract

**Background:**

Since older adults spend significant time in their neighborhood environment, environmental factors such as neighborhood socioeconomic disadvantage, high racial segregation, low healthy food availability, low access to recreation, and minimal social engagement may have adverse effects on cognitive function and increase susceptibility to dementia. DNA methylation, which is associated with neighborhood characteristics as well as cognitive function and white matter hyperintensity (WMH), may act as a mediator between neighborhood characteristics and neurocognitive outcomes.

**Methods:**

In this study, we examined whether DNA methylation in peripheral blood leukocytes mediates the relationship between neighborhood characteristics and cognitive function (*N* = 542) or WMH (*N* = 466) in older African American (AA) participants without preliminary evidence of dementia from the Genetic Epidemiology Network of Arteriopathy (GENOA).

**Results:**

For a 1-mile buffer around a participant’s residence, each additional fast food destination or unfavorable food store with alcohol per square mile was nominally associated with a 0.05 (95%CI: 0.01, 0.09) and a 0.04 (0.00, 0.08) second improvement in visual conceptual tracking score, respectively. Also, each additional alcohol drinking place per square mile was nominally associated with a 0.62 (0.05, 1.19) word increase in delayed recall score, indicating better memory function (all *p* < 0.05). Neighborhood characteristics were not associated with WMH. We did not find evidence that DNA methylation mediates the observed associations between neighborhood characteristics and cognitive function.

**Conclusions:**

The presence of fast food destinations and unfavorable food stores with alcohol was associated cognitive measures, possibly due to greater social interaction provided in these venues. However, replication of these findings is necessary. Further examination of the potential pathways between the neighborhood environment and cognitive function/WMH may allow the development of potential behavioral, infrastructural, and pharmaceutical interventions to facilitate aging in place and healthy brain aging in older adults, especially in marginal populations that are most at risk.

**Supplementary Information:**

The online version contains supplementary material available at 10.1186/s12916-024-03845-7.

## Background

Dementia is preceded by a noticeable decline in cognitive abilities that becomes severe enough to interfere with daily functioning [1]. Among US adults ages 65 and older, approximately 10% have dementia and 22% have mild cognitive impairment (MCI) [2]. Dementia, which includes Alzheimer’s disease (AD), vascular dementia (VaD), and other types of dementia, places a substantial burden on family, friends, and healthcare systems [3]. Cerebral small vessel disease (SVD), detected on magnetic resonance imaging (MRI) as white matter hyperintensities (WMH), causes one quarter of all ischemic strokes and is associated with cognitive function [4] and VaD [5–7]. To date, there are no effective treatments available to prevent or cure dementia. However, some research suggests that performing cognitively stimulating exercises and treating cardiovascular risk factors may delay or prevent the onset of dementia and reduce its associated pathology [1, 8]. While individual-level factors, such as educational attainment [9, 10], smoking habits [11], and physical activity [12, 13], are associated with cognitive function, there is growing interest in how neighborhood characteristics may shape health behaviors and health outcomes in older adults [14, 15].

Neighborhoods are defined as living environments that possess both physical and social attributes that may affect the health of their residents [16]. Specifically, characteristics of the neighborhood social environment, such as fewer destinations within walking distance that allow for social interaction and community, and low neighborhood socioeconomic status (SES) are associated with lower levels of cognitive function [17–20] and higher incidence of ischemic stroke [21, 22] in older adults. Since older adults spend a large proportion of their time in their neighborhood environment [23], factors such as neighborhood socioeconomic disadvantage [24], high racial segregation [25–28], low healthy food availability [29], low access to recreation [30, 31], and minimal social engagement [32] may have adverse effects on cognitive function and SVD and may also increase susceptibility to dementia [24–32]. As such, access to specific neighborhood infrastructures that may counteract these factors, such as increased access to healthy food, educational resources, safe and walkable neighborhoods, and recreational community activities, may support cognitive health among older adults aging in place [17]. Understanding how characteristics of neighborhood environments and how the accessibility of resources pertaining to healthy food and social and recreational activities impact the underlying molecular mechanisms of dementia pathology may allow us to develop better interventions to prevent disease onset.

Epigenetic modifications, such as DNA methylation, are molecular mechanisms that regulate gene expression without changing the underlying DNA sequence. DNA methylation, or the addition of a CH_3_ (methyl) group at a cytosine base followed by a guanine base (CpG site), is one of the most commonly studied epigenetic mechanisms. DNA methylation has been proposed as a potential mechanistic link between environmental exposures and downstream diseases because it is responsive to environmental stimuli, is dynamic across the life course, and is potentially reversible [33]. Given the regulatory role of DNA methylation on gene expression, as well as the association between CpG sites and aging [34], there has been a growing interest in understanding the extent to which DNA methylation contributes to age-related diseases such as Alzheimer’s and related dementia risk [35–39].

Previous studies have linked several individual- and neighborhood-level social disadvantage indicators, including low adult SES [35, 36] and living in disadvantaged neighborhoods [37–39], to DNA methylation patterns. After adjusting for individual SES, neighborhood socioeconomic disadvantage and social environment were also associated with DNA methylation in stress- and inflammation-related genes [38]. In addition, epigenome-wide association studies (EWAS) have shown associations between methylation and cognitive function [40, 41] and WMH [42, 43]. Since DNA methylation has been associated with both neighborhood-level factors and cognitive function/WMH, it may act as a mediator between neighborhood-level risk factors and cognitive outcomes. To date, a handful of studies have examined whether epigenome-wide markers mediate the effects of social disadvantage on health outcomes and risk factors. For example, in the New England Family Study, epigenetic markers from adipose tissue partially mediated the association between individual-level social disadvantage and body mass index in adulthood [44, 45]. In the Multi-Ethnic Study of Atherosclerosis (MESA), methylation from monocytes partially mediated the associations between adult SES and/or neighborhood socioeconomic disadvantage and several Cardiovascular disease risk factors [46]. To our knowledge, no studies have examined epigenetic mediation in the association between neighborhood characteristics and cognitive function/WMH.

African Americans (AA) have a greater burden of and risk for developing dementia [47–50] and stroke [51], compared to non-Hispanic Whites (NHW). Underlying causes of these disparities remain poorly understood but are likely due to multifactorial and multilevel factors that occur over the life-course. For example, differences in cognitive performance and dementia risk in AA may in part be caused by racial disparities in education (amount and quality) [52, 53], availability of material and social resources [54], access to favorable food and physical activity environments [55], exposure to discrimination [56], and neurotoxicants [57, 58]. A previous study in Atherosclerosis Risk in Communities (ARIC) found that reducing hypertension, obesity, and physical inactivity through targeted interventions could significantly lower dementia rates among Black individuals, particularly by addressing structural barriers to health [59]. While studies have examined individual-level risk factors as explanations for racial/ethnic disparities (e.g., socioeconomic, psychosocial, genetic, epigenetic, and biological), there is increasing interest in the role of the neighborhood on health outcomes in AA populations. Altogether, AA are more likely to live in neighborhoods with factors that may affect their stress levels (e.g., higher discrimination, lower educational attainment, and lower SES) that over time may result in physiological dysregulation [27] that ultimately leads to hypertension, diabetes, coronary heart disease, and depression. Dysregulation of neurocognitive processes may also lead to cognitive decline or dementia.

To better understand the mechanisms underlying relationships between neighborhood environment and dementia risk factors in older AA, we used high-dimensional mediation methods to identify DNA methylation sites (CpGs) in peripheral blood leukocytes that may mediate the relationship between neighborhood-level factors and cognitive function or WMH in the Genetic Epidemiology Network of Arteriopathy (GENOA) study.

## Methods

### Sample

The Genetic Epidemiology Network of Arteriopathy (GENOA) is a community-based longitudinal study intended to examine the genetic effects of hypertension and related target organ damage [60]. European American (EA) and African American (AA) hypertensive sibships were recruited if at least two siblings were clinically diagnosed with hypertension before age 60. All other siblings were invited to participate, regardless of hypertension status. Exclusion criteria included secondary hypertension, alcoholism or drug abuse, pregnancy, insulin-dependent diabetes mellitus, active malignancy, or serum creatinine levels > 2.5 mg/dL. Race/ethnicity was self-reported as non-Hispanic White or non-Hispanic Black. Next, genetic principal components (PCs) were used to confirm that the non-Hispanic Black participants clustered between European (CEU) and African (YOR) samples from 1000 Genomes Project Phase 1 (2012) [61], while non-Hispanic White participants clustered with European ancestry samples.

In phase I (1996–2001), 1854 AA participants (Jackson, MS) and 1583 EA participants (Rochester, MN) were recruited [60]. In phase II (2000–2004), 1482 participants AA participants and 1239 EA participants were successfully followed up, and their potential target organ damage from hypertension was measured. Demographics, medical history, clinical characteristics, medication use, and blood samples were collected in each phase. Methylation levels were measured only in AA participants using blood samples collected in phases I and II.

In an ancillary study, the Genetics of Microangiopathic Brain Injury (GMBI; 2001–2006), 1010 AA and 967 EA GENOA participants underwent a battery of established cognitive tests to assess measures of cognitive function [62, 63]. White matter hyperintensity (WMH) was also measured using brain magnetic resonance imaging (MRI) for the majority of GMBI participants. The GMBI exam occurred approximately 1 year after the participant completed phase II (mean time between phase II and GMBI = 1.1 years, SD = 1.0 year). Written informed consent was obtained from all participants, and approval was granted by participating institutional review boards (University of Michigan, University of Mississippi Medical Center, and Mayo Clinic).

After excluding participants with missing neurocognitive test data (*n* = 93) and neighborhood density measures (*n* = 4), we had a total of 913 AA participants with available demographic, cognitive, and neighborhood data (Additional File 1: Fig. S1). Since participants with a history of stroke or dementia may have had changes in general cognitive function that differed from non-pathological cognitive aging, we excluded those with a history of stroke (*n* = 40) and/or preliminary evidence of dementia indicated by a Mini-Mental State Examination Score (MMSE) of < 24 (*n* = 52). Participants younger than age 45 were also excluded (*n* = 96). After further excluding participants missing genetic PCs (*n* = 183), a total of 542 and 477 participants were available with neighborhood spatial (density measures) and neighborhood socioeconomic disadvantage analyses, respectively (Additional File 1: Fig. S1). For WMH analyses, a total of 466 and 404 participants were available for neighborhood spatial (density measures) and neighborhood socioeconomic disadvantage analyses, respectively (Additional File 1: Fig. S2).

### Measures

#### Measures of cognitive function

The following four cognitive domains were evaluated: delayed recall (Rey Auditory Verbal Learning Test [RAVLT]), processing speed (Digit Symbol Substitution Test [DSST]), word fluency (Controlled Oral Word Association Test [COWA-FAS]), and visual conceptual tracking (Trail Making Test A [TMTA]) [62–64]. All cognitive domains were coded so that a higher score corresponds to better cognitive function. See Additional File 2: Supplementary Methods for details.

In addition to analyzing individual cognitive domains, we assessed a summary measure of general cognitive function, which is often quantified using cognitive tests in multiple cognitive domains [65]. In this study, general cognitive function was calculated as the first unrotated principal component (FUPC) from a principal component analysis (PCA) of the four cognitive domains in the full sample (*N* = 542). The FUPC accounted for 57% of the total variance in the cognitive measures and loading factors of the four measures were 0.61 for delayed recall (RAVLT), 0.88 for processing speed (DSST), 0.70 for word fluency (COWA-FAS), and 0.81 for visual conceptual tracking (TMTA).

#### White matter hyperintensity

Presence of WMH in brain samples indicates areas of ischemic damage to small vessels and surrounding areas. Brain magnetic resonance images were measured from magnetic resonance imaging (MRI), using Signa 1.5 T MRI scanners (GE Medical Systems, Waukesha, WI, USA) at Mayo Clinic [66]. For additional details, see Smith et al. [67] WMH and total brain volume in the corona radiata and periventricular zone were quantified from axial fluid-attenuated inversion recovery (FLAIR) images [68]. Brain scans with cortical infarctions were excluded from the analyses because of the distortion of WMH volume estimates that would be introduced in the automated segmentation algorithm. Models assessing WMH were adjusted for total intracranial volume (TIV). Distributional plots indicated that the measures of WMH are right-skewed, so the WMH variable was transformed as ln(WMH + 1).

#### DNA methylation

Genomic data was extracted from stored peripheral blood leukocytes from 1106 AA GENOA participants from phase I and 304 AA participants from phase II using the AutoGen FlexStar (AutoGen, Holliston, MA). Bisulfite conversion was performed with the EZ DNA Methylation Kit (Zymo Research, Irvine, CA), and methylation was measured using the Illumina HumanMethylationEPIC BeadChip. The raw intensity data was visualized using the shinyMethyl R package [69] to identify sex mismatches and outliers, which were removed. Samples with incomplete bisulfite conversion were identified using Qcinfo in the *Enmix* R package [70] and removed. Background correction and dye-bias normalization were performed using Noob in the *Minfi* R package [71, 72]. Sample identity was verified using 59 SNP probes on the EPIC array, and mismatched samples were removed. Probe-type bias was adjusted using the Regression on Correlated Probes (RCP) method [73]. Probes with detection *p*-value < 10^−16^ were considered successfully detected, and probes and samples with detection rate < 10% were removed [74]. We also excluded cross-reactive probes [75] and probes with a SNP at the target CpG site or within a single-base extension. After quality control, a total of 1396 samples (*N* = 1100 from phase I and *N* = 294 from phase II) and 857,121 CpG sites were available for analysis. For this analysis, all methylation data were from phase I samples. White blood cell proportions for CD8 + T lymphocytes, CD4 + T lymphocytes, natural killer cells, B cells, monocytes, and granulocytes were estimated using the Houseman method [76]. For each CpG site prior to analysis, the methylation beta-values [77, 78] were pre-adjusted for batch effects (sample plate, row, and column) and white blood cell proportions using linear mixed modeling, and the resulting residuals were added to the mean values.

#### Genotype data

Genetic PCs were estimated from genotype data obtained from the Illumina HumanOmni2.5 arrays, as previously described [79].

#### Individual-level measures

Age was assessed at cognitive testing. The respondent’s highest level of educational attainment was categorized as (1) less than high school degree/GED (reference group), (2) high school degree or GED, and (3) at least 4 years of college or trade/technical school. Smoking has a substantial impact on the epigenome [80], so we used smoking data from the same timepoint as the DNA methylation measures (phase I). Participants were categorized as current, former, or never smokers (reference group).

## Neighborhood characteristics

### GIS-based measures

Densities of neighborhood destinations were derived from the National Establishment Time Series (NETS) [81] data (1996–2015). Simple densities per square mile were created for ½-mile, 1-mile, and 3-mile buffer sizes around home addresses of GENOA participants at phase I using ArcGIS V.9.3 (ESRI, Inc., Redlands, California) [82–84]. We used 1-mile buffer in our primary analysis, as previous studies have done [85, 86], and examined ½- and 3-mile buffers in sensitivity analysis. Kernel densities per square mile, with greater weighting towards destinations located closer to the home of a participant, were also created for GENOA participants using the kernel density command in ArcGIS V.9.3 [82–84] for the same buffer sizes; these were also explored in sensitivity analysis.

For each participant, simple densities were estimated for the following 10 types of destinations: fast food restaurants (including both chain and non-chain), total physical activity facilities, total social engagement destinations, alcohol outlets, unfavorable food stores with and without alcohol, healthy (favorable) food stores, popular walking destinations, and total food stores. The modified retail food environment index (MRFEI) was also calculated from the number of healthy and less healthy food retailers within census tracts across states, based on typical food offerings in specific retail stores [87–89]. See Additional File 2: Supplementary Methods for details.

### Census measures

Briefly, neighborhood socioeconomic disadvantage was assessed using data collected in the 2000 US Census [90, 91] and American Community Survey (ACS) 2005–2009 [92, 93]. Data was linked to GENOA participant data (phase I; 1995–2000) by census tract using Census and ACS estimates for the closest time period. To derive neighborhood socioeconomic disadvantage, we used six variables that reflected aspects of wealth and income, education, and occupation for each census tract [94]. *Z*-scores for each census tract were estimated for each variable, and neighborhood socioeconomic disadvantage was defined as the sum of *Z*-scores from the six variables, with higher scores indicating more disadvantage. See Additional File 2: Supplementary Methods for details.

### Statistical analysis

We first calculated Pearson correlations among the six outcomes (general cognitive function, the four cognitive domains and WMH) and among the 13 neighborhood characteristics (12 density measures and neighborhood socioeconomic disadvantage). Since areas of increased population density (e.g., urban neighborhoods) generally have a higher absolute number of destinations, we next examined the neighborhood characteristics after pre-adjusting for census tract population density using linear modeling. Correlations were calculated among the neighborhood characteristics for simple and kernel densities per square mile for 1-mile buffer sizes.

#### Associations between neighborhood measures and cognitive function/WMH

To identify which exposures and outcomes have a significant total effect, we tested for association between each neighborhood characteristic (exposure) and general cognitive function, each cognitive domain, or WMH (outcome). We first tested for association between a neighborhood characteristic (socioeconomic disadvantage or simple density measures) and general cognitive function, adjusting for age at cognitive function measurement, sex, current smoking status, the first 5 genetic PCs of ancestry, and family relatedness as a random effect (model 1a). While PCs are likely not a confounder of the relationship between neighborhood and cognitive function/WMH, we included them so that we would have the same adjustment variables in the total effects model as in the mediation models (described below) when we next examine methylation as a mediator of the relationship between neighborhood and cognitive function/WMH. In model 1b, we tested for association between each neighborhood characteristic and WMH, adjusting for the same covariates as model 1a and TIV. In models 2a/2b, we additionally adjusted for census tract population density in 2000 and included census tract as a random effect. We also tested for associations between each neighborhood characteristic and each of the four cognitive domains using model 2a. Associations between neighborhood characteristics and cognitive function/WMH that were significant at *p* < 0.05 in models 1a/1b or 2a/2b were selected for mediation analysis. In sensitivity analysis, we tested the same associations using simple densities at ½- and 3-mile buffers as well as kernel densities at all 3 buffers. Because we were interested in identifying total effects to investigate further under the hypothesis that methylation is a mediator of these relationships, we were interested in any associations meeting a nominal significance level (*p* < 0.05). However, since we conducted a large number of tests, we also assessed whether any were significant after multiple testing using false discovery rate (FDR *q* < 0.01) [95]. The total effects model is outlined below:

$${Y}_{2jk}={\beta }_{0}+ \omega {X}_{1jk}+ \boldsymbol{\alpha }{C}_{1jk}+{W}_{k}+{\varepsilon }_{jk}$$

${\beta }_{0}$: intercept value; cognitive function/WMH value when all covariates (neighborhood characteristic (exposure) and confounders) equal zero.

$\omega$: effect estimate of neighborhood characteristic (exposure) on cognitive function/WMH.

${X}_{1jk}$: neighborhood characteristic (exposure) for participant j in sibship k at phase I.

${C}_{1jk}$: set of covariates (age at cognitive function/WMH measurement, sex, and genetic principal components at phase I and TIV for WMH outcome).

${W}_{k}$: random effect (familial relatedness; independent and normal distribution) in sibship k.

${\varepsilon }_{jk}$: residual error (independent and normal distribution) for participant j in sibship k.

${Y}_{2jk}$: cognitive function/WMH for participant j in sibship k at phase II.

#### Mediation analysis

If a significant association (total effect) was identified between a neighborhood characteristic and a cognitive/WMH outcome, we conducted an epigenome-wide high-dimensional mediation analysis to identify CpG sites that may partially mediate the relationship. We used a cross-product-based mediation approach in which the mediation effect is obtained by multiplying the exposure-mediator effect (*β*_*1*_) and the mediator-outcome effect (*β*_*3*_; see Eqs. 1 and 2). We obtained these parameters for each exposure and outcome tested using linear mixed models to separately estimate the association between neighborhood characteristics with DNA methylation (mediator), while adjusting for covariates (Eq. 1), and the association between DNA methylation and cognitive function/WMH, while adjusting for the corresponding exposure tested and the same set of covariates (Eq. 2). The covariate sets in Eqs. 1 and 2 are the same as in models 1a/b and 2a/b. The specified models (Eqs. 1 and 2) for a given exposure-outcome association are outlined below:

1 $${M}_{jk}={\beta }_{0}+ {\beta }_{1}{X}_{1jk}+\boldsymbol{\alpha }{V}_{1jk}+{W}_{k}+{\varepsilon }_{jk}$$

2 $${Y}_{2jk}= {\beta }_{0}+{\beta }_{2}{X}_{1jk}+{\beta }_{3}{M}_{jk}+\boldsymbol{\alpha }{V}_{1jk}+{W}_{k}+{\varepsilon }_{jk}$$

${\beta }_{0}$: intercept value; cognitive function/WMH value when all covariates (neighborhood characteristic (exposure) and confounders) equal zero.

${M}_{jk}$: DNA methylation (mediator; beta-value) for participant *j* in sibship *k.*

${X}_{1jk}$: neighborhood characteristic (exposure) for participant *j* in sibship *k* at phase I.

${V}_{1jk}$: adjustment covariates for participant *j* in sibship *k* at phase I.

${W}_{k}$: random effect (familial relatedness; independent and normal distribution) in sibship *k.*

${\varepsilon }_{jk}$: residual error (independent and normal distribution) for participant *j* in sibship *k.*

${Y}_{2jk}$: cognitive function/WMH (outcome) for participant *j* in sibship *k* at phase II.

*β*_*1*_*:* effect estimate of neighborhood characteristic (exposure) on DNA methylation (mediator).

*β*_*2*_*:* direct effect estimate of the neighborhood characteristic (exposure) on cognitive function/WMH (outcome).

*β*_*3*_*:* effect estimate of DNA methylation (mediator) on cognitive function/WMH (outcome), adjusting for the direct effect (*β*_*2*_).

Using Eqs. 1 and 2, the epigenetic mediation effect was tested using the following:

H_0_: $${\beta }_{1}{\beta }_{3}$$ = 0.

H_A_: $${\beta }_{1}{\beta }_{3}$$ ≠ 0.

**Fig. 1:**
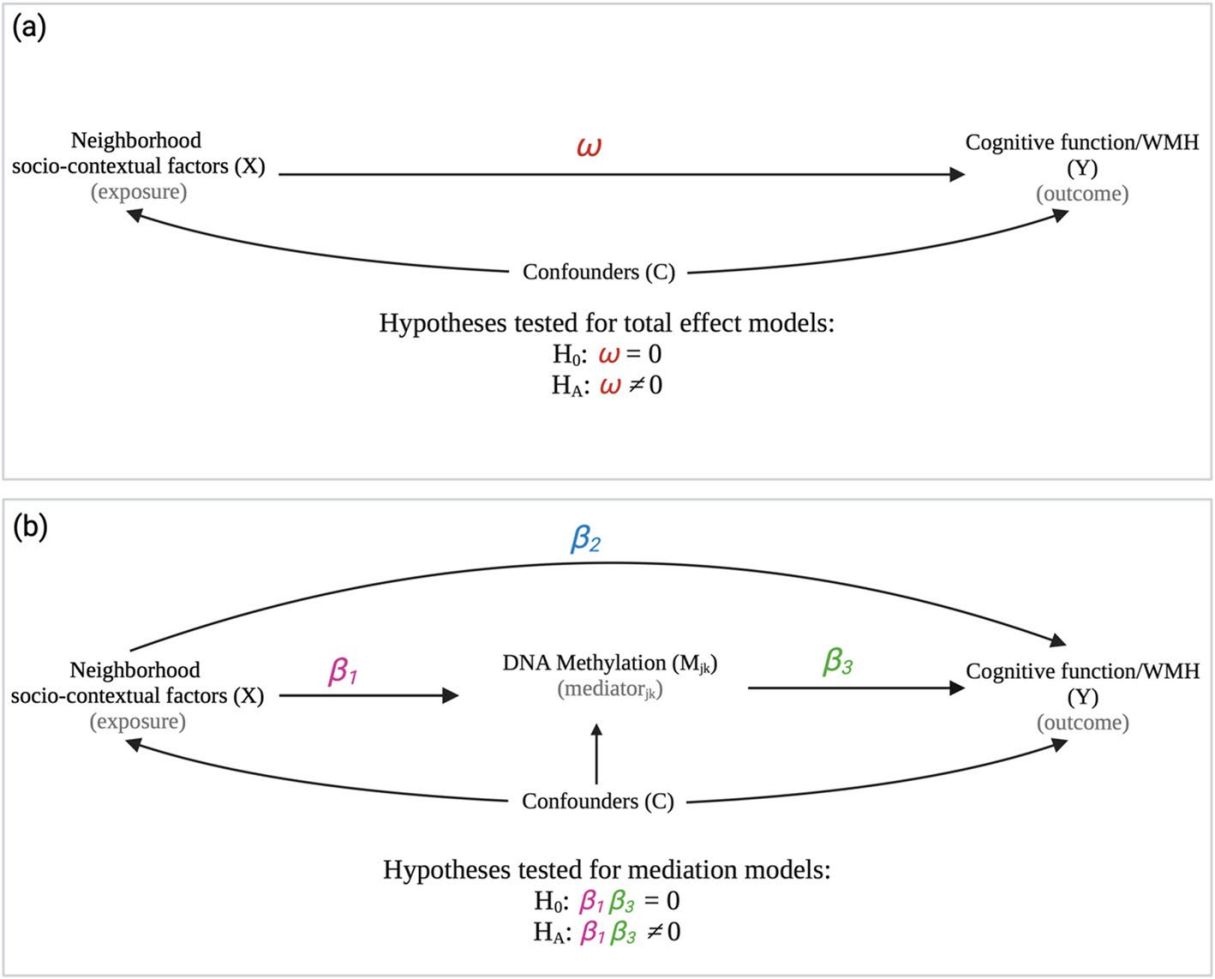
Directed acyclic graph (DAG) of the hypothesized associations for the epigenetic mediation between neighborhood characteristics (exposures) and cognitive/WMH outcomes. **a** The total effect associations between neighborhood characteristic (X) and cognitive function/WMH (Y). $\omega$ is the effect estimate of the neighborhood characteristic on cognitive function/WMH. **b** The mediation effect obtained through the cross-product-based mediation approach obtained by multiplying the exposure-mediator effect (*β*_*1*_) and the mediator-outcome effect (*β*_*3*_). Confounders (C) include age at measurement, PCs 1–4, sex, education, smoking status, familial relatedness, neighborhood socioeconomic disadvantage, census tract population density, and census tract (model 2a/2b only)

The null hypothesis was comprised of three sub-hypotheses: (1)* H*_*01*_: ${\beta }_{1}=0,$ ${\beta }_{3}\ne 0$; (2)* H*_*10*_: ${\beta }_{1}\ne 0,$ ${\beta }_{3}=0$; and (3)* H*_*00*_: ${\beta }_{1}={\beta }_{3}=0$. We performed parallel one-at-a-time mediation hypothesis testing. With a total of *M* mediators, we denote π_01_, π_10_, and π_00_ as the true proportions of (${\beta }_{1}=0,$ ${\beta }_{3}\ne 0)$, (${\beta }_{1}\ne 0,$ ${\beta }_{3}=0$), and $\left({\beta }_{1}={\beta }_{3}=0\right)$ among all *M* tests. Figure 1 shows a directed acyclic graph (DAG) of the hypothesized associations. To test for the mediation effect, we used the Sobel-comp [96] method in the *medScan* package in R, which uses a corrected mixture reference distribution for Sobel’s test statistic according to the composite structure of the null hypothesis. We considered *p* < 0.05 to be significant.

## Results

### Sample characteristics

**Fig. 2:**
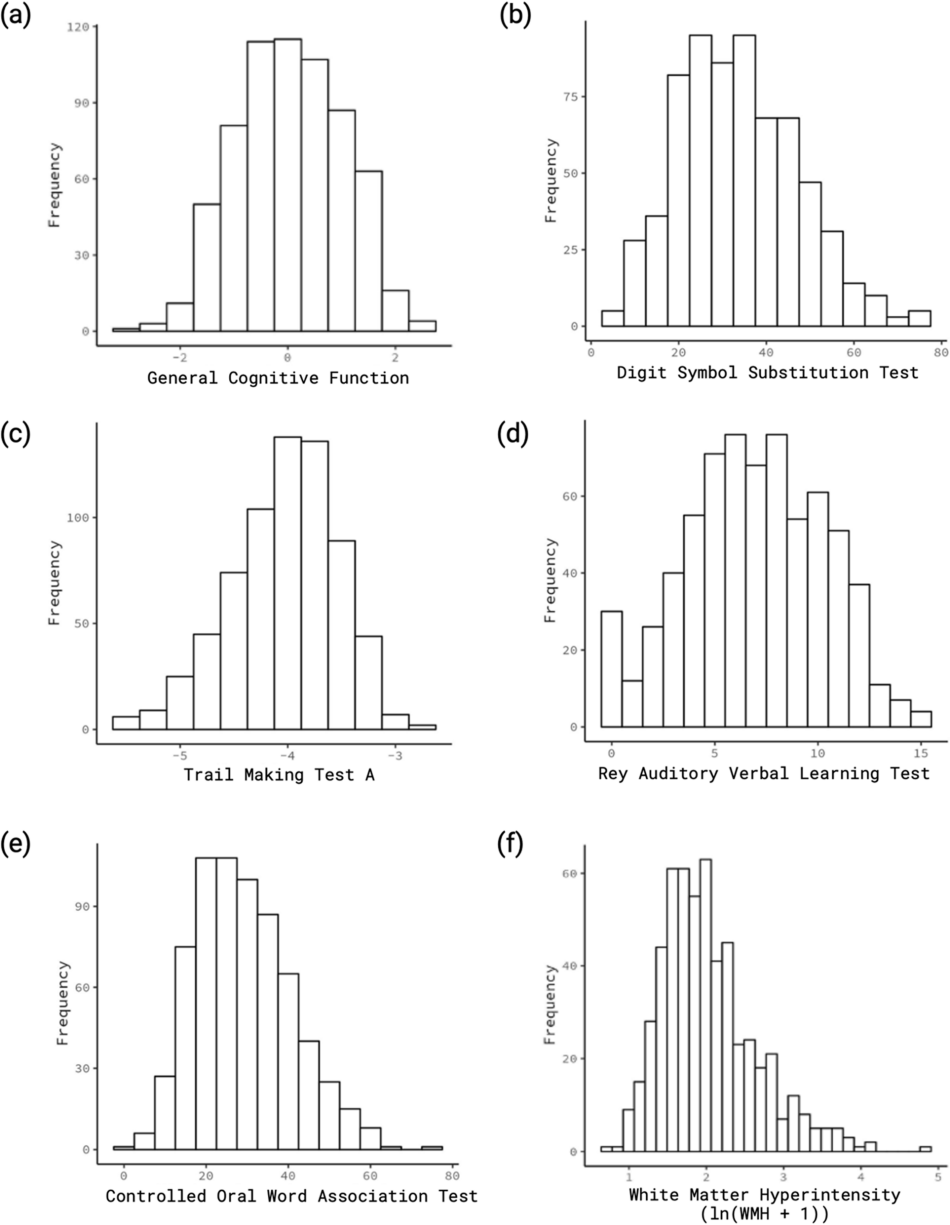
Distributions of cognitive and structural brain measures. **a** General cognitive function, **b** Digit Symbol Substitution Test, **c** Trail Making Test A, **d** Rey Auditory Verbal Learning Test, **e** Controlled Oral Word Association Test, and **f** log-transformed white matter hyperintensity (ln(WMH + 1))

The sample included 542 AA without dementia (Table 1). Participant age ranged from 45 to 83 years (mean = 62.5 years). More than half of participants (73%) were female. A total of 25.0% had less than a high school degree/GED, 46.5% attained a high school degree/GED, and 28.6% completed at least 4 years of college or trade school. General cognitive function was normally distributed (Fig. 2). Mean delayed recall (RAVLT) score was 7.0 (SD = 3.3) words recalled, mean processing speed (DSST) was 33.8 (SD = 13.0) symbols, mean word fluency (COWA-FAS) score was 29.4 (SD = 11.6) words, and mean visual conceptual tracking (TMTA) score was 63.8 (SD = 35.2) seconds to completion. Participants had a mean WMH of 9.42 cm^3^ (SD = 9.19). WMH distribution was severely right skewed but had a normal distribution after log transformation (Fig. 2).

**Table 1:** Sample characteristics of Genetic Epidemiology Network of Arteriopathy (GENOA) African Americans (*N* = 542)

	Mean (SD) or *n*%
Age at cognition measurement (years)	62.52 (7.69)
Sex	
Female	403 (74.35%)
Male	139 (25.65%)
Educational attainment	
Completed at least 4 years of college or technical/trade school	155 (28.60%)
Completed high school degree/GED	252 (46.49%)
Less than high school degree/GED	135 (24.91%)
Smoking status	
Current smoker	83 (15.31%)
Former smoker	125 (23.06%)
Never smoker	334 (61.62%)
General cognitive function	0.03 (0.99)
Delayed recall (RAVLT, number of words recalled)	6.95 (3.29)
Processing speed (DSST, number of symbols)	33.82 (13.04)
Word fluency (COWA-FAS, number of words)	29.40 (11.64)
Visual conceptual tracking (TMTA, seconds to test completion)	63.75 (35.22)
White matter hyperintensity (WMH, cm^3^)^a^	9.42 (9.19)
Total intracranial volume (TIV, cm^3^)^a^	1376.58 (129.81)
Neighborhood characteristics	
Neighborhood socioeconomic disadvantage	3.41 (3.46)
Fast food destination density^b^	0.75 (0.85)
Unfavorable food stores without alcohol density^b^	1.94 (1.75)
Unfavorable food stores with alcohol density^b^	1.24 (1.13)
Favorable food stores density^b^	0.22 (0.31)
Total physical activity destinations density^b^	0.34 (0.37)
Total social engagement destinations density^b^	14.37 (10.85)
Total popular walking destination density^b^	3.53 (3.13)
Alcoholic drinking places density^b^	0.36 (0.62)
Total food stores density^b^	3.34 (3.08)
MRFEI with alcohol^c^	0.10 (0.13)
MRFEI without alcohol^c^	0.12 (0.14)

*Abbreviations*: *RAVLT*, Rey Auditory Verbal Learning Test; *DSST*, Digit Symbol Substitution Test; *COWA-FAS*, Controlled Oral Word Association Test; *TMTA*, Trail Making Test A; *WMH*, white matter hyperintensity; *MRFEI*, Modified Retail Food Environment Index

^a^Sample size = 466

^b^Simple density measures per square mile for 1-mile buffer size

^c^Derived from simple density measures per square mile for 1-mile buffer size

### Correlation among cognitive and WMH outcomes

The four cognitive domains were moderately correlated (Pearson *r* ranged from 0.21 to 0.68), with the highest correlation among processing speed (DSST) and visual conceptual tracking (TMTA) (*r* = 0.68, *p* < 0.001, Additional File 3: Table S1). WMH was negatively and weakly correlated with all the cognitive measures except COWA-FAS (Pearson *r* ranged from − 0.27 to − 0.34 for significant correlations).

### Correlation among the neighborhood exposures

Neighborhood exposures were moderately correlated (Pearson *r* ranged from − 0.24 to 0.99, Additional File 3: Table S2). Neighborhood socioeconomic disadvantage was positively, but weakly, correlated with unfavorable food stores without alcohol, total social engagement destinations, total popular walking destinations, and alcoholic drinking places. After adjusting for census tract population density, the correlations between neighborhood socioeconomic disadvantage and neighborhood characteristics increased in magnitude in the positive direction for all measures except fast food destinations, alcoholic drinking places, and the MRFEI measures (Additional File 3: Tables S3 and S4).

### Associations between neighborhood characteristics and cognitive/WMH outcomes

#### Neighborhood socioeconomic disadvantage associations

Neighborhood socioeconomic disadvantage was not associated with general cognitive function or WMH either before (models 1a/1b) or after adjusting for census tract population density and census tracts as a random effect (models 2a/2b, Table 2). Furthermore, neighborhood socioeconomic disadvantage was not associated with any of the four cognitive domains (model 2a, Table 3).

**Table 2:** Associations between neighborhood socioeconomic disadvantage and cognition/WMH

	General cognitive function (*N* = 477)				White matter hyperintensity (*N* = 404)				
	Model 1a		Model 2a		Model 1b			Model 2b	
	*β* (95% CI)	*p*	*β* (95% CI)	*p*	*β* (95% CI)	*p*	*β* (95% CI)		*p*
Neighborhood socioeconomic disadvantage	− 0.01 (− 0.03, 0.01)	0.30	− 0.01(− 0.03, 0.01)	0.36	2.0E − 3 (− 0.01, 0.02)	0.83	0.01 (− 0.01, 0.03)		0.28

*Abbreviations*: *CI* confidence interval, *WMH* white matter hyperintensity, *PC* principal component

Model 1a: cognitive function = neighborhood socioeconomic disadvantage + age at measurement + sex + PC1-4 + education + smoking status + familial relatedness (random effect)

Model 2a: cognitive function = model 1a + census tract population density + census tract (random effect)

Model 1b: WMH = model 1a + total intracranial volume

Model 2b: WMH = model 2a + total intracranial volume

^*^p < 0.05

**Table 3:** Associations between neighborhood socioeconomic disadvantage and cognitive measures (model 2a; *N* = 477)

	DSST		COWA-FAS		RAVLT		TMTA	
	*β* (95% CI)	*p*	*β* (95% CI)	*p*	*β* (95% CI)	*p*	*β* (95% CI)	*p*
Neighborhood socioeconomic disadvantage	− 0.01 (− 0.38, 0.36)	0.95	0.02 (− 0.33, 0.37)	0.92	− 0.03 (− 0.14, 0.09)	0.66	0.02 (− 0.03, 0.00)	0.07

*Abbreviations*: *DSST*, Digit Symbol Substitution Test; *COWA-FAS*, Controlled Oral Word Association Test; *RAVLT*, Rey Auditory Verbal Learning Test; *TMTA*, Trail Making Test A; *CI*, confidence interval

Model 2a: neurocognitive measure = neighborhood socioeconomic disadvantage + age at measurement + sex + PC1-4 + education + smoking status + population density + familial relatedness (random effect) + census tract (random effect)

^*^p < 0.05

#### Density associations

There was no association between the 12 neighborhood simple density exposures at 1-mile buffer size and cognitive/WMH outcomes either before (models 1a/1b) or after adjusting for census tract population density and census tracts as a random effect (models 2a/2b; Table 4). The associations between simple neighborhood densities per square mile for ½- and 3-mile buffer sizes and cognitive function/WMH are reported in Additional File 3: Table S5. One additional alcoholic drinking place per square mile for the 3-mile buffer size was nominally associated with a 0.71 SD (95% CI: − 1.38, − 0.04) decrease in general cognitive function after adjusting for census tract population density and census tracts as a random effect (*p* = 0.03; model 2a; Additional File 3: Table S5). However, after multiple testing correction, no associations were significant (all FDR *q* > 0.1).

**Table 4:** Associations between simple density of neighborhood destinations per square mile for 1-mile buffer size and cognitive function/WMH

Neighborhood characteristics	General cognitive function				White matter hyperintensity			
	Model 1a (*N* = 542)		Model 2a (*N* = 477)		Model 1b (*N* = 466)		Model 2b (*N* = 404)	
	*β* (95% CI)	*p*	*β* (95% CI)	*p*	*β* (95% CI)	*p*	*β* (95% CI)	*p*
Fast food destination density	− 0.02 (− 0.09, 0.05)	0.53	− 0.03 (− 0.11, 0.05)	0.39	0.03 (− 0.03, 0.09)	0.23	0.04 (− 0.02, 0.10)	0.25
Unfavorable food stores without alcohol density	− 0.02 (− 0.06, 0.02)	0.38	− 0.02 (− 0.06, 0.02)	0.37	0.01 (− 0.02, 0.04)	0.40	0.02 (− 0.02, 0.06)	0.24
Unfavorable food stores with alcohol density	− 0.03 (− 0.09, 0.03)	0.26	− 0.05 (− 0.11, 0.01)	0.14	0.02 (− 0.02, 0.06)	0.26	0.03 (− 0.01, 0.07)	0.25
Favorable food stores density	− 0.08 (− 0.28, 0.12)	0.45	− 0.11 (− 0.33, 0.11)	0.31	0.02 (− 0.13, 0.17)	0.83	− 0.01 (− 0.17, 0.15)	0.84
Total physical activity destinations density	− 0.07 (− 0.23, 0.09)	0.36	− 0.05 (− 0.25, 0.15)	0.58	0.03 (− 0.10, 0.16)	0.65	0.05 (− 0.09, 0.19)	0.58
Total social engagement destinations density	− 3.16E − 03 (− 0.01, 0.00)	0.29	− 3.59E − 03 (0.00, 0.00)	0.35	1.59E − 03 (0.00, 0.01)	0.49	3.46E − 03 (0.00, 0.00)	0.24
Total popular walking destination density	− 3.75E − 03 (− 0.02, 0.02)	0.71	− 2.49E − 03 (− 0.02, 0.02)	0.84	0.01 (− 0.01, 0.03)	0.38	0.01 (− 0.01, 0.03)	0.25
Alcoholic drinking places density	− 0.01 (− 0.11, 0.09)	0.78	0.01 (− 0.11, 0.13)	0.89	1.86E − 03 (− 0.08, 0.08)	0.99	0.03 (− 0.07, 0.13)	0.52
Total food stores density	− 0.01 (− 0.03, 0.01)	0.63	− 3.80E − 03 (− 0.02, 0.02)	0.77	2.21E − 03 (− 0.01, 0.02)	0.78	0.01 (− 0.01, 0.03)	0.37
Modified Retail Food Environment Index with alcohol	− 0.10 (− 0.70, 0.50)	0.73	− 0.13 (− 0.76, 0.50)	0.69	0.17 (− 0.25, 0.59)	0.41	0.08 (− 0.37, 0.53)	0.74
Modified Retail Food Environment Index without alcohol	− 0.02 (− 0.55, 0.51)	0.93	− 0.05 (− 0.62, 0.52)	0.85	0.10 (− 0.28, 0.48)	0.58	0.03 (− 0.40, 0.46)	0.90

*Abbreviations*: *CI* confidence interval, *WMH* white matter hyperintensity, *PC* principal component

Model 1a: cognitive function = neighborhood characteristic + age at measurement + PC1-4 + sex + education + smoking status + familial relatedness (random effect)

Model 2a: cognitive function = model 1a + neighborhood socioeconomic disadvantage + census tract population density (random effect) + census tract (random effect)

Model 1b: WMH = model 1a + total intracranial volume

Model 2b: WMH = model 2a + total intracranial volume

^*^p < 0.05; **p < 0.0021 (i.e., *p* < 0.05 after Bonferroni correction for 24 tests)

We also tested the association between the 12 neighborhood simple density exposures examined at 1-mile buffer region with each of the four cognitive domains (model 2a; Table 5). One additional fast food destination or unfavorable food store with alcohol per square mile was nominally associated with a 0.05 (95% CI: 0.01, 0.09; *p* = 0.04) and a 0.04 (95% CI: 0.00, 0.08; *p* = 0.04) second increase in visual conceptual tracking score, respectively, indicating that more of these destinations was associated with better visual conceptual tracking. In addition, one additional alcohol drinking place per square mile was nominally associated with a 0.62 word (95% CI: 0.05, 1.19; *p* = 0.03) increase in delayed recall score (Table 5), indicating better memory function. However, no associations were significant at FDR *q* < 0.1. The associations between simple neighborhood densities per square mile for ½- and 3-mile buffer sizes and cognitive/WMH measures are reported in Additional File 3: Tables S5 and S6.

**Table 5:** Associations between simple density of neighborhood destinations per square mile for 1-mile buffer size and cognitive measures (model 2a; *N* = 477)

Neighborhood characteristics	DSST		COWA-FAS		RAVLT		TMTA	
	*β* (95% CI)	*p*	*β* (95% CI)	*p*	*β* (95% CI)	*p*	*β* (95% CI)	*p*
Fast food destination density	− 0.39 (− 1.45, 0.67)	0.45	0.27 (− 0.87, 1.41)	0.63	0.1 (− 0.27, 0.47)	0.57	0.05 (0.01, 0.09)	0.04*
Unfavorable food stores without alcohol density	− 0.17 (− 0.74, 0.40)	0.55	− 0.19 (− 0.80, 0.42)	0.52	0.13 (− 0.07, 0.33)	0.18	0.02 (0.00, 0.04)	0.19
Unfavorable food stores with alcohol density	− 0.45 (− 1.29, 0.39)	0.28	− 0.07 (− 0.97, 0.83)	0.87	0.01 (− 0.28, 0.30)	0.94	0.04 (0.00, 0.08)	0.04*
Favorable food stores density	− 1.46 (− 4.36, 1.44)	0.30	0.2 (− 2.90, 3.30)	0.89	− 0.31 (− 1.29, 0.67)	0.52	0.12 (− 0.02, 0.26)	0.08
Total physical activity destinations density	− 1.07 (− 3.60, 1.46)	0.39	− 1.18 (− 3.88, 1.52)	0.37	0.44 (− 0.42, 1.30)	0.30	0.05 (− 0.07, 0.17)	0.38
Total social engagement destinations density	− 0.06 (− 0.16, 0.04)	0.26	− 0.03 (− 0.13, 0.07)	0.61	0.02 (− 0.02, 0.06)	0.25	0.00 (0.00, 0.00)	0.36
Total popular walking destination density	− 0.05 (− 0.38, 0.28)	0.77	0.02 (− 0.33, 0.37)	0.88	0.09 (− 0.01, 0.19)	0.09	0.01 (− 0.01, 0.03)	0.20
Alcoholic drinking places density	0.16 (− 1.51, 1.83)	0.85	− 0.93 (− 2.67, 0.81)	0.28	0.62 (0.05, 1.19)	0.03*	− 3.11E − 03 (− 0.08, 0.08)	0.94
Total food stores density	− 0.01 (− 0.36, 0.34)	0.95	− 0.11 (− 0.48, 0.26)	0.53	0.02 (0.00, 0.04)	0.07	0.01 (− 0.01, 0.03)	0.41
Modified Retail Food Environment Index with alcohol	− 3.56 (− 11.55, 4.43)	0.36	4.15 (− 4.43, 12.73)	0.32	0.1 (− 0.02, 0.22)	0.65	0.20 (− 0.19, 0.59)	0.28
Modified Retail Food Environment Index without alcohol	− 3.29 (− 10.54, 3.96)	0.36	4.43 (− 3.37, 12.23)	0.25	− 0.64 (− 3.48, 2.20)	0.66	0.20 (− 0.13, 0.53)	0.21

*Abbreviations*: *DSST *Digit Symbol Substitution Test, *COWA-FAS* Controlled Oral Word Association Test, *RAVLT* Rey Auditory Verbal Learning Test, *TMTA* Trail Making Test A, *CI* confidence interval, *PC* principal component

Model 2a: neurocognitive measure = neighborhood characteristic + age at measurement + PC1-4 + sex + education + smoking status + neighborhood socioeconomic disadvantage + census tract population density + familial relatedness (random effect) + census tract (random effect)

^*^p < 0.05; **p < 0.001 (i.e., *p* < 0.05 after Bonferroni correction for 48 tests)

We also tested the associations between the 12 neighborhood kernel density exposures at ½-, 1- and 3- mile buffer sizes with cognitive function/WMH (Additional File 3: Table S7) and the cognitive domains (Additional File 3: Table S8). There were no associations between the kernel density neighborhood exposures and general cognitive function or WMH in models 1a/2a and 1b/2b (Additional File 3: Table S7). At the 1-mile buffer, kernel density of fast food destinations and unfavorable food stores with alcohol were both nominally associated with better visual conceptual tracking, consistent with the simple density associations; however, the association between kernel density of alcohol drinking places and delayed recall score was not. We also found that at the 1-mile buffer, kernel densities of unfavorable food stores without alcohol, total popular walking destinations, and total food stores were all nominally associated with better visual conceptual tracking as well. However, no associations were significant at FDR *q* < 0.1. The associations between kernel neighborhood densities per square mile for ½- and 3-mile buffer sizes and cognitive/WMH measures are also reported in Additional File 3: Tables S7 and S8.

### Mediation analysis

**Fig. 3:**
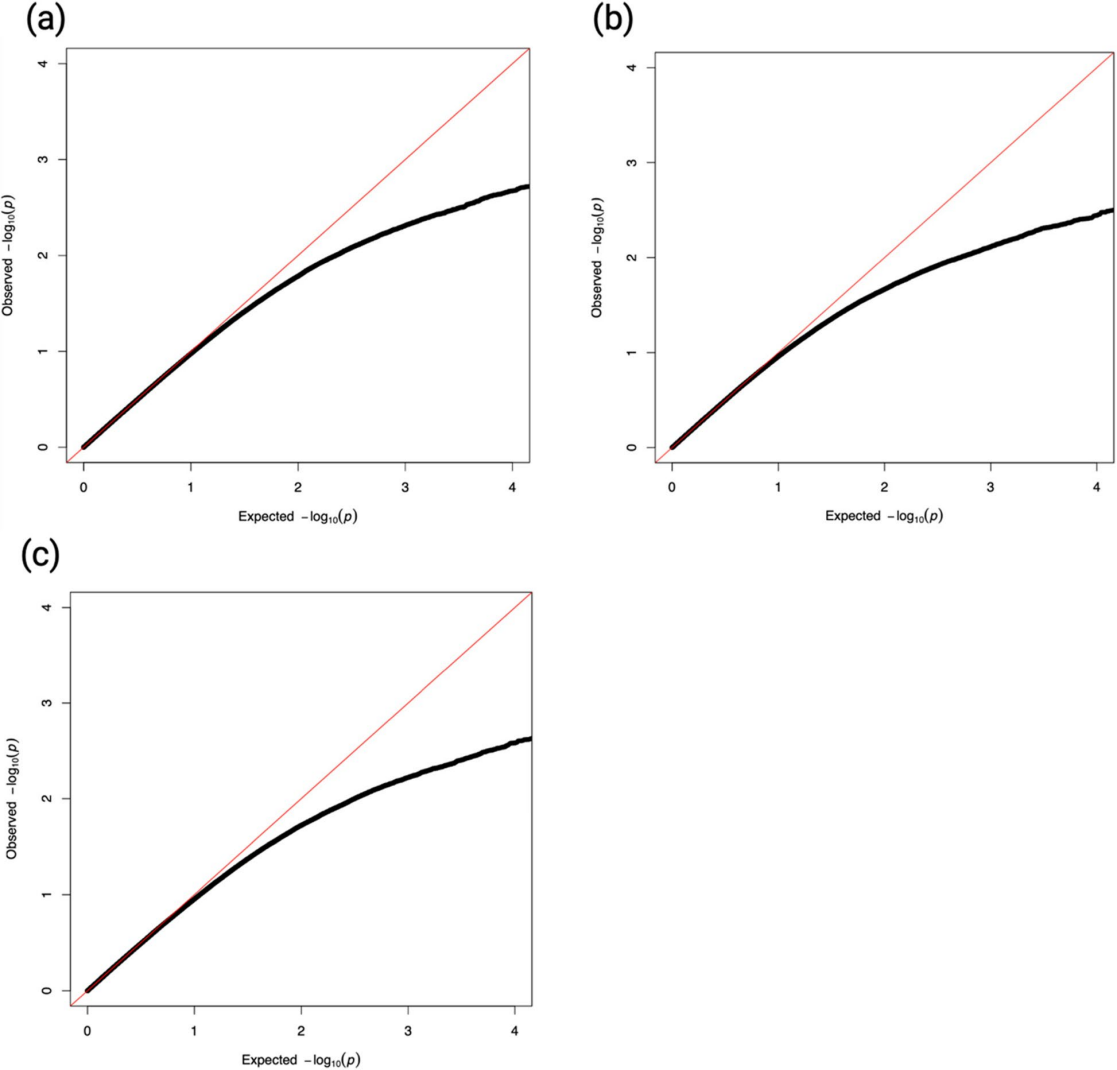
Quantile–quantile (QQ) plots for the epigenetic mediation of the associations between neighborhood characteristics and cognitive function. QQ plots for the Sobel-Comp mediation hypothesis testing method with *N* = 477 observations. The exposures are simple densities per square mile for 1-mile buffer sizes, the outcomes are neurocognitive measures, and the mediators are 857,121 CpG sites. The exposure–outcome models tested are as follows: **a** alcohol drinking places density—RAVLT, **b** fast food destination density—TMTA, and **c** unfavorable food stores (with alcohol) density—TMTA. Mediation models are adjusted for age, sex, education, smoking status, first four principal components, neighborhood socioeconomic disadvantage, and census tract population density, with family and census tracts as random effects

When the total effect of a neighborhood characteristic (simple density at 1-mile buffer) and cognitive function/WMH was significant at *p* < 0.05, we conducted epigenome-wide high-dimensional mediation analysis to identify possible CpG sites that may partially mediate the relationship between the neighborhood exposure and corresponding outcome using model 2a in 477 participants with complete data. The following exposure-outcome combinations were investigated: (a) alcohol drinking places and delayed recall, (b) fast food destinations and visual conceptual tracking, and (c) unfavorable food stores with alcohol and visual conceptual tracking. Figure 3 shows quantile–quantile (QQ) plots for the 5 exposure-outcome relationships using Sobel-Comp. The *p*-values from Sobel-Comp test were deflated, potentially due to the large number of zero exposure-mediator (*β*_*1*_) and mediator-outcome (*β*_*3*_) estimates and the small sample size (Fig. 3).

## Discussion

As the aging population rapidly grows, a better understanding of how the neighborhood environment may affect cognitive health is needed to mitigate the future burden of dementia in the USA. While there are studies showing the effect of individual factors, such as lifestyle, genetics and biomarkers on cognitive function, there is a need for more research on the association between neighborhood characteristics and cognitive function to date [97]. Furthermore, only a few studies have examined the potential molecular mechanisms linking neighborhood environment and cognitive health [17, 98]. To our knowledge, this study is the first assessment of whether DNA methylation partially mediates the association between various neighborhood environment characteristics and cognitive function in AA without dementia. This cross-sectional study suggests that greater simple densities of alcohol drinking places may be associated with better memory as measured by delayed recall (RAVLT) and greater densities of fast food destination and unfavorable food stores with alcohol with better attention and processing speed as measured by visual conceptual tracking (TMTA) in cognitively normal AA. However, we did not find associations between neighborhood characteristics and WMH. We also were unable to detect mediating effects of DNA methylation on the associations between these neighborhood characteristics on cognitive function and cognitive measures in this sample. Nevertheless, our findings that neighborhood density of fast food restaurants and bars may serve as a protective resource rather than as a risk factor challenge status quo public health paradigms and are an important contribution that shows the potential utility of community and third places.

We initially expected higher densities of unfavorable food stores to be associated with worse cognitive function, suggesting that increased access to unhealthy food and drink may encourage unhealthy dietary choices that lead to lower cognitive health. Instead, we found that greater densities of alcohol drinking places, fast food, and unfavorable stores with alcohol that may encourage unhealthy dietary choices were associated with better cognitive function as measured by delayed recall and visual conceptual tracking after adjustment for population density. Considering that Jackson, MS, does not have a highly dense population (approximately 1300 people per square mile in 2010), the presence of these walking destinations may provide meeting places for community members, allowing for greater interaction and stimulation of cognitive health, regardless of their impact on unhealthy diet and behaviors. As such, these meeting hubs may contribute to better cognitive function through increased access to community residents, neighborhood community resources, and proximal walking destinations that improve cognitive health by increasing physical activity levels, social engagement, mental health or quality of life [99].

To date, results from previous studies examining similar characteristics of the neighborhood environment and cognitive function have been mixed. In the Chicago Health and Aging Project (CHAP), increasing densities of social and walking destinations such as community centers were associated with slower cognitive decline [100], yet a study in the Multi-Ethnic Study of Atherosclerosis (MESA) showed an inverse association between these same measures and cognitive function and most noticeably in individuals of non-white race [101]. Also, closer access to community resources has been associated with better cognitive function in NHW, but worse cognitive function in AA [102], while other studies showed no association between the presence of neighborhood built environment characteristics, such as recreation centers and institutional resources (e.g., libraries, schools and community centers) and cognitive function [100, 102, 103]. In our study, the plausible mechanisms and direction or presence of neighborhood-cognitive function association may depend on the neighborhood characteristic and cognitive domain being studied, and more than one mechanism may be at play.

Different underlying mechanisms of neighborhood environment on cognitive function have been examined to understand how interventions can prevent dementia onset. In MESA, increasing social destination density, walking destination density, and intersection density were associated with worse cognitive function, and increasing proportion of land dedicated to retail was associated with better processing speed [104]. While we did not observe similar patterns among simple densities, we did observe greater kernel densities of total popular walking destinations per square mile (for ½- and 1-mile buffer sizes) were associated with higher visual conceptual tracking and greater kernel densities of total social engagement destinations per square mile (½-mile buffer) were associated with higher delayed recall. Access to a safe and walkable neighborhood environment may help older adults age in place and delay the onset of cognitive impairment and decline prior to dementia [103, 105, 106]. In addition, the positive relationship between proportion of land dedicated to retail and processing speed may be explained by increased utilitarian physical activity and social engagement or increased cognitive stimulation that contributes to the cognitive reserve [103]. Also, fast food outlets and local retail food environments may play a role in providing social and community engagement, connectedness, emotional support, and cognitive stimulation for older adults outside of more formal or age-graded settings such as doctor’s office, church, or senior center [107, 108].

Other studies have found inverse relationships between neighborhood destinations (such as retail stores) and cognitive function that may be related to cognitive overload among older adults due to stress from greater number of destination choices or navigation of traffic. It is possible that highly dense areas consisting of social and walking destinations and street intersections have increased vehicular pollutant exposure due to decreased distances to busy roadways and decreased air ventilation created by buildings [109]. Airborne pollutants have been associated with worse cognitive function and brain structure in older adults [109]. Neighborhood factors such as low SES, high racial segregation, and unhealthy diet and lifestyle habits may increase susceptibility to cognitive decline and dementia [24–32]. These mixed results from other studies may be affected by residual confounding from unmeasured factors. Thus, additional research on the many confounders and mechanisms related to the relationship between the neighborhood environment and cognitive function is necessary.

In addition, we found correlations between favorable and unfavorable destinations, even after adjusting for population density, which may further illuminate our findings in the context of cognitive health and behaviors. For example, greater densities of fast food destinations were associated with greater densities of favorable food stores, physical activity destinations, and MRFEI (the proportion of favorable food stores to total food stores), even after adjusting for population density. These correlations in Jackson may be attributed to a complex interplay of socioeconomic, urban planning, cultural, historical, and policy-related factors and confounders. Furthermore, socioeconomic disparities often lead to variations in access to health-promoting resources, with neighborhoods of lower SES facing limited access to healthy options and an increased prevalence of unhealthy alternatives. The availability of favorable food stores may reflect the demand from residents, according to their purchasing power, who can afford healthier options. To account for this discrepancy, we adjusted for neighborhood socioeconomic disadvantage in our associations. The city’s urban planning, historical development (e.g., redlining and discriminatory housing practices in the past), and government policies may play crucial roles in shaping the distribution of health-related destinations. Another possibility is that areas with higher commercial zoning may attract both fast food establishments and favorable food stores, creating clusters of businesses in certain neighborhoods. Additionally, cultural preferences and consumer demand influence the types of businesses and amenities in specific neighborhoods. For example, the high correlation between favorable and unfavorable food store density may be due to a micro-cultural artifact at play in Jackson that encourages increased densities of fast food in Black neighborhoods [110]. This micro-culture, which results from shared race/ethnicity, beliefs, styles, skills, and habits of residents of a particular area, may disfavor physical activity and other healthy behaviors, even in the presence of features that allow for them [111, 112].

To date, the relationships between neighborhood disadvantage markers and health outcomes in AA have been mixed. Multiple theories have been proposed to describe minority communities that have been historically oppressed and their reliance on community-specific, and often non-institutional, resources [113]. For example, the “weathering” hypothesis of racial inequality proposes that Black individuals endure early health deterioration due to cumulative economic and social disadvantages across the life course [114]. As such, multiple and chronic stressors may result in wear and tear on health from an increased “allostatic load.” Another theory of “cognitive reserve” proposes that other cognitive attributes may compensate for cognitive health in the case that other faculties (e.g., brain tissue integrity) are weakened [115]. Lastly, based on Marginalized-Related Diminished Returns (MDRs), there may be no innate or neurobiological explanation for observed racial disparities; instead, adverse social factors (e.g., structural racism, segregated schools, poor education and social disparities such as unsafe neighborhoods) may prevent Black communities (across socioeconomic levels) from securing tangible gains from their higher educational attainment [116–118]. To that end, it is important to consider the effects of structural racism, social stratification, Jim Crow, redlining and racial segregation on Black communities when considering cognitive aging and other health disparities. In this study, results are mixed; however, they present the possibility that third places and gathering spaces among community may be important for the overall cognitive health of AA. As such, while genetic factors may play some role in explaining racial disparities in health between AA and EA, social factors may be more important [119, 120]. Effective efforts and interventions to reduce chronic stressors and improve health treatments would not only focus on the individual but must also seek to alter the social, economic, and political structures that cause disease in vulnerable populations [119]. Further research is warranted at the intersection of race, SES, and cognitive health, as the racial disparities in the effects of risk and protective factors for dementia has been understudied [57].

Considering that the neighborhood context has the potential to influence cognitive function, it is important to clarify the potential biological mechanisms linking neighborhood characteristics and cognitive function to shed light on the etiology and causal mechanisms driving health disparities. DNA methylation may help us better understand the pathways that mediate or interact with the environment and cognitive function. Previous studies have shown that the neighborhood context affects DNA methylation, even after adjusting for individual- level factors and that DNA methylation patterns in stress and inflammatory pathways may be responsive to interventions [38]. EWAS have also found multiple CpGs related to neurodegeneration associated with cognitive function [40, 41]. Considering these factors and that past studies have found CpGs mediating the relationship between neighborhood socioeconomic disadvantage and various CVD risk factors [44–46], which are potential upstream factors of cognitive function and dementia, we expected to detect mediating CpG sites in the associations between neighborhood characteristics and cognitive function/WMH.

One reason that we may not have observed epigenetic mediation is because genetic factors may play a smaller role in cognitive function for AA than NHW. For example, the strongest risk factor for dementia, *APOE* epsilon 4, has a weaker effect in AA than Whites [121, 122]. Perhaps neighborhood factors also impact cognitive function through pathways outside of genetic changes. A second reason could be the choice of mediation model implemented. Sobel-Comp [96] is a more powerful extension of high-dimensional mediation hypothesis testing (HDMT) [123] that is preferred when almost all exposure-mediator and mediator-outcome associations are equal to 0 (π_00_ is close to 1), and there are almost no non-zero exposure-mediator or mediator-outcome associations (π_01_ and π_10_ are close to 0). One limitation is that Sobel-Comp is conservative under these conditions, compared to other high-dimensional mediation methods such as JT-Comp [124]; however, Sobel-Comp has the advantages of using the correct mixture reference distribution for Sobel’s test statistic, maintaining a false positive rate (FPR) close to the nominal level, and it yielding larger true positive rates (TPRs). In this study, Sobel-Comp was the appropriate method because π_00_ was bounded away from 1 for all associations tested, but we did not detect significant mediation effects due to a potentially large number of zero exposure-mediator (*β*_*1*_) and mediator-outcome (*β*_*3*_) estimates, deflated *p*-values, and small sample size. In addition, DNA methylation levels of proximal CpGs in the same biological pathways may be correlated, resulting in properties that are not desirable for TPR and FPR [77]. When there are correlated mediators, single-mediator hypothesis testing methods like Sobel-Comp are unable to fully account for all the mediator-outcome confounders affected by the exposure (also known as co-mediators), thus reducing the power to detect mediating CpGs and potentially biasing our effect estimates [46, 125–127]. While it is possible to jointly model multiple mediators using the Bayesian high-dimensional mediation method [128] and its use may have reduced the multiple testing burden and increased the power to detect independent effects, this method is computationally heavy and only a few thousand mediators would have been evaluated simultaneously [128–130]. Evaluating our mediation analysis models to account for multiple correlated mediators are of interest for future analysis. Our results may indicate that methylation is not a critical component of the mediating pathway between neighborhood exposures and cognitive/WMH outcomes.

Our observed associations should also be considered with caution due to the limited statistical power inherent in our sample. The small sample size may have restricted our ability to detect the total effects between neighborhood characteristics and cognitive/WMH outcomes that could exist within the population. In addition, although our total effect associations allowed us to begin to characterize the relationships between neighborhood factors and cognitive function, findings did not reach statistical significance when accounting for multiple testing using FDR, which could be attributed to small sample size and power. Research using AA samples with larger sample sizes is needed to better understand how neighborhood characteristics are related to cognitive/WMH outcomes in AA populations.

Our study also had other limitations. Our findings may be affected by residual confounding by unmeasured variables, increased exposure to factors including air pollution, potential for chance social interactions, crime, physical disability, discrimination, and structural racism that may be due to increased walking in the neighborhood which influences cognitive function, or factors related to study design (e.g., cross-sectional nature, bias due to loss-to-follow-up, or bias due to refusal of blood draw). Notably, GENOA is a unique example in that it was initially established to recruit hypertensive sibships. As such, participants were already actively engaging with the University of Mississippi for their medical care and part of a research knowledgeable community that was more likely to trust doctors and be a part of the medical system. In addition, participants were accessing family resources for their hypertension status and other potential comorbidities, indicating that the cohort is possibly sicker than those in the general population. As such, our cohort may have intrinsic selection bias to the nature of those living in the area and having agreed to be a part of the GENOA study. Moreover, we did not investigate the important ways in which air pollution, structural racism and stress are mediators on the pathways of specific neighborhood-cognitive function/WMH associations. Also, further longitudinal and life-course studies that explore mediation pathways between early-life, mid-life, and late-life neighborhood, methylation, and cognitive function/WMH measures are needed. In this study, neighborhood characteristics were based on current home addresses, and we did not take into account that earlier or longer-term neighborhood exposures may be important for late-life cognitive function/WMH.

Our study also has notable strengths. To our knowledge, this study is the first to examine the role of DNA methylation in mediating the relationships between neighborhood characteristics and cognitive function/WMH in a cohort of older adults without diagnosed dementia. Our study was also conducted in AA, an understudied population with a higher prevalence of dementia [131, 132] and higher conferred risk of cognitive decline and dementia from neighborhood environment compared to EA [133]. Additionally, with rich cognitive and WMH measures, we were able to assess associations with multiple cognitive domains, general cognitive function, and a risk factor for VaD. We were also able to adjust for neighborhood socioeconomic disadvantage to control for the influence of income, education, employment, and other SES indicators that might independently affect cognitive health. We also controlled for confounding by census tract population density because it could influence the availability of stores and cognitive outcomes. High-density urban areas may have greater access to stores and services, and low-density rural areas may have lower access to these destinations. Both densities may affect cognitive health, so adjusting for population density ensures that our results are not skewed by these population differences and are more accurate. Also, we utilized a powerful high dimensional mediation method that reduced the likelihood of false positives. Lastly, our primary analysis used 1-mile density buffers around participants’ homes, which provide more precise spatial representation of neighborhoods than administrative boundaries and may more accurately reflect nearby places and distances that an older adult would walk.

## Conclusions

In the present study, we found that destination density had small but notable effects on several domains of cognitive function in AA without dementia. However, we detected no significant mediating effects of DNA methylation on these associations. Upon further examination of the potential pathways between the neighborhood environment and cognitive function, we may develop potential behavioral, infrastructural, and pharmaceutical interventions to allow aging in place and healthy brain aging in older adults, especially marginal populations that are most at risk.

## Supplementary Information

Additional File 1: Fig. S1. Flow diagram illustrating sample sizes for neighborhood density and neighborhood socioeconomic disadvantage analyses for cognitive measures in GENOA AA. Fig. S2. Flow diagram illustrating sample sizes for neighborhood density and neighborhood socioeconomic disadvantage analyses for white matter hyperintensity in GENOA AA.

Additional File 2: Supplementary Methods

Additional File 3: Table S1. Pearson’s correlations among the cognitive/WMH outcomes (*N* = 466). Table S2. Pearson’s correlations among neighborhood socioeconomic disadvantage and neighborhood simple density measures per square mile for 1-mile buffer size (*N* = 542). Table S3. Associations among neighborhood socioeconomic disadvantage and neighborhood simple density measures per square mile for 1-mile buffer size after adjusting for census tract population density (*N*= 542). Table S4. Pearson’s correlations among neighborhood socioeconomic disadvantage and simple and kernel densities per square mile for 1-mile buffer size. Table S5. Associations between simple density of neighborhood destinations per square mile for ½-, 1- and 3- mile buffer sizes and cognitive function/WMH. Table S6. Associations between simple density of neighborhood destinations per square mile for ½-, 1- and 3- mile buffer sizes and cognitive measures (*N* = 477). Table S7. Associations between kernel density of neighborhood destinations per square mile for ½-, 1- and 3- mile buffer sizes and cognitive function/WMH. Table S8. Associations between kernel density of neighborhood destinations per square mile for ½-, 1- and 3- mile buffer sizes and cognitive measures (*N* = 477).

## Data Availability

The phenotype data used in the current study are available upon reasonable request to J.A.S. and S.L.R.K. and with a completed data use agreement (DUA). Genotype data are available from the Database of Genotypes and Phenotypes (dbGaP): *Genetic Epidemiology Network of Arteriopathy (GENOA)*, accession number: phs001238.v2.p1, https://www.ncbi.nlm.nih.gov/projects/gap/cgi-bin/study.cgi?study_id=phs001238.v2.p1, released 08/08/2018. Methylation data are available from the Gene Expression Omnibus (GEO): *Methylation data from stored peripheral blood leukocytes from African American participants in the GENOA study*, accession number: GSE210256, https://www.ncbi.nlm.nih.gov/geo/query/acc.cgi?acc=GSE210256, released 08/04/2022. Due to IRB restriction, mapping of the sample IDs between genotype data (dbGaP) and methylation data (GEO) cannot be provided publicly but is available upon written request to J.A.S. and S.L.R.K.

## Abbreviations

AA: African American
AD: Alzheimer’s disease
COWA-FAS: Controlled Oral Word Association Test
CpG: Cytosine-phosphate-guanine
DAG: Directed acyclic graph
DNAm: DNA methylation
DSST: Digit Symbol Substitution Test
EA: European Americans
EWAS: Epigenome-wide association study
FDR: False discovery rate
FLAIR: Fluid-attenuated inversion recovery
FUPC: First unrotated principal component
GENOA: Genetic Epidemiology Network of Arteriopathy
GMBI: Genetics of Microangiopathic Brain Injury
MCI: Mild cognitive impairment
MMSE: Mini Mental State Exam
MRI: Magnetic resonance imaging
NHW: Non-Hispanic whites
PC: Principal component
PCA: Principal component analysis
RA: Risk allele
RAF: Risk allele frequency
RAVLT: Rey Auditory Verbal Learning Test
SCWT: Stroop Color-Word Test
SD: Standard deviation
SES: Socioeconomic status
SVD: Small vessel disease
TMTA: Trail Making Test A
VaD: Vascular dementia
WMH: White matter hyperintensity

## References

[1] Vemuri P, Lesnick TG, Przybelski SA, Knopman DS, Machulda M, Lowe VJ, et al. Effect of intellectual enrichment on AD biomarker trajectories: Longitudinal imaging study. Neurology. 2016;86(12):1128–35. 26911640 10.1212/WNL.0000000000002490 PMC4820132

[2] Manly JJ, Jones RN, Langa KM, Ryan LH, Levine DA, McCammon R, et al. Estimating the prevalence of dementia and mild cognitive impairment in the US: the 2016 Health and Retirement Study Harmonized Cognitive Assessment Protocol Project. JAMA Neurology. 2022 Oct 24 [cited 2022 Nov 10]; Available from: 10.1001/jamaneurol.2022.3543 10.1001/jamaneurol.2022.3543 PMC9593315 36279130

[3] Hurd MD, Martorell P, Delavande A, Mullen KJ, Langa KM. Monetary costs of dementia in the United States. N Engl J Med. 2013;368(14):1326–34. 23550670 10.1056/NEJMsa1204629 PMC3959992

[4] Kuller LH, Shemanski L, Manolio T, Haan M, Fried L, Bryan N, et al. Relationship between ApoE, MRI findings, and cognitive function in the Cardiovascular Health Study. Stroke. 1998;29(2):388–98. 9472879 10.1161/01.str.29.2.388

[5] Markus HS, Hunt B, Palmer K, Enzinger C, Schmidt H, Schmidt R. Markers of endothelial and hemostatic activation and progression of cerebral white matter hyperintensities. Stroke. 2005;36(7):1410–4. 15905468 10.1161/01.STR.0000169924.60783.d4

[6] Williams OA, Zeestraten EA, Benjamin P, Lambert C, Lawrence AJ, Mackinnon AD, et al. Predicting dementia in cerebral small vessel disease using an automatic diffusion tensor image segmentation technique. Stroke. 2019;50(10):2775–82. 31510902 10.1161/STROKEAHA.119.025843 PMC6756294

[7] Prins ND, Scheltens P. White matter hyperintensities, cognitive impairment and dementia: an update. Nat Rev Neurol. 2015;11(3):157–65. 25686760 10.1038/nrneurol.2015.10

[8] Nelson L, Tabet N. Slowing the progression of Alzheimer’s disease; what works? Ageing Res Rev. 2015;1(23):193–209. 10.1016/j.arr.2015.07.002 26219494

[9] Aneshensel CS, Ko MJ, Chodosh J, Wight RG. The urban neighborhood and cognitive functioning in late middle age. J Health Soc Behav. 2011;52(2):163–79. 21673145 10.1177/0022146510393974 PMC3152319

[10] Wight RG, Aneshensel CS, Miller-Martinez D, Botticello AL, Cummings JR, Karlamangla AS, et al. Urban neighborhood context, educational attainment, and cognitive function among older adults. Am J Epidemiol. 2006;163(12):1071–8. 16707655 10.1093/aje/kwj176

[11] Nooyens ACJ, van Gelder BM, Verschuren WMM. Smoking and cognitive decline among middle-aged men and women: the Doetinchem Cohort Study. Am J Public Health. 2008;98(12):2244–50. 18923116 10.2105/AJPH.2007.130294 PMC2636537

[12] Lautenschlager NT, Almeida OP. Physical activity and cognition in old age. Curr Opin Psychiatry. 2006;19(2):190–3. 16612202 10.1097/01.yco.0000214347.38787.37

[13] Eckstrom E, Neukam S, Kalin L, Wright J. Physical activity and healthy aging. Clin Geriatr Med. 2020;36(4):671–83. 33010902 10.1016/j.cger.2020.06.009

[14] Sallis JF, Saelens BE, Frank LD, Conway TL, Slymen DJ, Cain KL, et al. Neighborhood built environment and income: examining multiple health outcomes. Soc Sci Med. 2009;68(7):1285–93. 19232809 10.1016/j.socscimed.2009.01.017 PMC3500640

[15] Koohsari MJ, Badland H, Sugiyama T, Mavoa S, Christian H, Giles-Corti B. Mismatch between perceived and objectively measured land use mix and street connectivity: associations with neighborhood walking. J Urban Health. 2015;92:242–52. 25539783 10.1007/s11524-014-9928-x PMC4411311

[16] Diez Roux AV, Mair C. Neighborhoods and health. Ann N Y Acad Sci. 2010;1186(1):125–45. 20201871 10.1111/j.1749-6632.2009.05333.x

[17] Besser LM, McDonald NC, Song Y, Kukull WA, Rodriguez DA. Neighborhood environment and cognition in older adults: a systematic review. Am J Prev Med. 2017;53(2):241–51. 28455123 10.1016/j.amepre.2017.02.013 PMC5522645

[18] Rosso AL, Flatt JD, Carlson MC, Lovasi GS, Rosano C, Brown AF, et al. Neighborhood socioeconomic status and cognitive function in late life. Am J Epidemiol. 2016;183(12):1088–97. 27257114 10.1093/aje/kwv337 PMC4908209

[19] Lang IA, Llewellyn DJ, Langa KM, Wallace RB, Huppert FA, Melzer D. Neighborhood deprivation, individual socioeconomic status, and cognitive function in older people: analyses from the English Longitudinal Study of Ageing. J Am Geriatr Soc. 2008;56(2):191–8. 18179489 10.1111/j.1532-5415.2007.01557.x PMC2671806

[20] Shih RA, Ghosh-Dastidar B, Margolis KL, Slaughter ME, Jewell A, Bird CE, et al. Neighborhood socioeconomic status and cognitive function in women. Am J Public Health. 2011;101(9):1721–8. 21778482 10.2105/AJPH.2011.300169 PMC3154213

[21] Lisabeth L, Diez Roux A, Escobar J, Smith M, Morgenstern L. Neighborhood environment and risk of ischemic stroke: the brain attack surveillance in Corpus Christi (BASIC) Project. Am J Epidemiol. 2007;165(3):279–87. 17077168 10.1093/aje/kwk005

[22] Brown AF, Liang LJ, Vassar SD, Stein-Merkin S, Longstreth WT Jr, Ovbiagele B, et al. Neighborhood disadvantage and ischemic stroke: the Cardiovascular Health Study (CHS). Stroke. 2011;42(12):3363–8. 21940966 10.1161/STROKEAHA.111.622134 PMC3781011

[23] Yen IH, Michael YL, Perdue L. Neighborhood environment in studies of health of older adults: a systematic review. Am J Prev Med. 2009;37(5):455–63. 19840702 10.1016/j.amepre.2009.06.022 PMC2785463

[24] Hunt JFV, Vogt NM, Jonaitis EM, Buckingham WR, Koscik RL, Zuelsdorff M, et al. Association of neighborhood context, cognitive decline, and cortical change in an unimpaired cohort. Neurology. 2021;96(20):e2500–12. 33853894 10.1212/WNL.0000000000011918 PMC8205478

[25] Pohl DJ, Seblova D, Avila JF, Dorsman KA, Kulick ER, Casey JA, et al. Relationship between residential segregation, later-life cognition, and incident dementia across race/ethnicity. Int J Environ Res Public Health. 2021;18(21):11233. 34769752 10.3390/ijerph182111233 PMC8583156

[26] Jang JB, Hicken MT, Mullins M, Esposito M, Sol K, Manly JJ, et al. Racial segregation and cognitive function among older adults in the United States: findings from the Reasons for Geographic and Racial Differences in Stroke Study. J Gerontol B Psychol Sci Soc Sci. 2022;77(6):1132–43. 34137853 10.1093/geronb/gbab107 PMC9159056

[27] Forrester SN, Gallo JJ, Whitfield KE, Thorpe RJ. A framework of minority stress: from physiological manifestations to cognitive outcomes. Gerontologist. 2019;59(6):1017–23. 30169640 10.1093/geront/gny104 PMC6858824

[28] Schulz A, Northridge ME. Social determinants of health: implications for environmental health promotion. Health Educ Behav. 2004;31(4):455–71. 15296629 10.1177/1090198104265598

[29] Auchincloss AH, Moore KA, Moore LV, Diez Roux AV. Improving retrospective characterization of the food environment for a large region in the United States during a historic time period. Health Place. 2012;18(6):1341–7. 22883050 10.1016/j.healthplace.2012.06.016 PMC3501601

[30] Powell LM, Chaloupka FJ, Slater SJ, Johnston LD, O’Malley PM. The availability of local-area commercial physical activity-related facilities and physical activity among adolescents. Am J Prev Med. 2007;33(4 Suppl):S292-300. 17884577 10.1016/j.amepre.2007.07.002

[31] Gordon-Larsen P, Nelson MC, Page P, Popkin BM. Inequality in the built environment underlies key health disparities in physical activity and obesity. Pediatrics. 2006;117(2):417–24. 16452361 10.1542/peds.2005-0058

[32] Finlay J, Esposito M, Li M, Kobayashi LC, Khan AM, Gomez-Lopez I, et al. Can neighborhood social infrastructure modify cognitive function? A mixed-methods study of urban-dwelling aging Americans. J Aging Health. 2021;33(9):772–85. 34301156 10.1177/08982643211008673 PMC8922945

[33] McGowan PO, Szyf M. The epigenetics of social adversity in early life: implications for mental health outcomes. Neurobiol Dis. 2010;39(1):66–72. 20053376 10.1016/j.nbd.2009.12.026

[34] Gopalan S, Carja O, Fagny M, Patin E, Myrick JW, McEwen LM, et al. Trends in DNA methylation with age replicate across diverse human populations. Genetics. 2017;206(3):1659–74. 28533441 10.1534/genetics.116.195594 PMC5500158

[35] Needham BL, Smith JA, Zhao W, Wang X, Mukherjee B, Kardia SLR, et al. Life course socioeconomic status and DNA methylation in genes related to stress reactivity and inflammation: the multi-ethnic study of atherosclerosis. Epigenetics. 2015;10(10):958–69. 26295359 10.1080/15592294.2015.1085139 PMC4844216

[36] Stringhini S, Polidoro S, Sacerdote C, Kelly RS, van Veldhoven K, Agnoli C, et al. Life-course socioeconomic status and DNA methylation of genes regulating inflammation. Int J Epidemiol. 2015;44(4):1320–30. 25889032 10.1093/ije/dyv060

[37] Giurgescu C, Nowak AL, Gillespie S, Nolan TS, Anderson CM, Ford JL, et al. Neighborhood environment and DNA methylation: implications for cardiovascular disease risk. J Urban Health. 2019;96(1):23–34. 30635842 10.1007/s11524-018-00341-1 PMC6430282

[38] Smith JA, Zhao W, Wang X, Ratliff SM, Mukherjee B, Kardia SLR, et al. Neighborhood characteristics influence DNA methylation of genes involved in stress response and inflammation: the Multi-Ethnic Study of Atherosclerosis. Epigenetics. 2017;12(8):662–73. 28678593 10.1080/15592294.2017.1341026 PMC5687339

[39] Lei MK, Beach SRH, Simons RL, Philibert RA. Neighborhood crime and depressive symptoms among African American women: genetic moderation and epigenetic mediation of effects. Soc Sci Med. 2015;146:120–8. 26513121 10.1016/j.socscimed.2015.10.035 PMC4655437

[40] Marioni RE, McRae AF, Bressler J, Colicino E, Hannon E, Li S, et al. Meta-analysis of epigenome-wide association studies of cognitive abilities. Mol Psychiatry. 2018;23(11):2133–44. 29311653 10.1038/s41380-017-0008-y PMC6035894

[41] McCartney DL, Hillary RF, Conole ELS, Banos DT, Gadd DA, Walker RM, et al. Blood-based epigenome-wide analyses of cognitive abilities. Genome Biol. 2022;23(1):26. 35039062 10.1186/s13059-021-02596-5 PMC8762878

[42] Ammous F, Zhao W, Ratliff SM, Kho M, Shang L, Jones AC, et al. Epigenome-wide association study identifies DNA methylation sites associated with target organ damage in older African Americans. Epigenetics. 2021;16(8):862–75. 33100131 10.1080/15592294.2020.1827717 PMC8331005

[43] Yang Y, Knol MJ, Wang R, Mishra A, Liu D, Luciano M, et al. Epigenetic and integrative cross-omics analyses of cerebral white matter hyperintensities on MRI. Brain. 2022;awac290. 10.1093/brain/awac290 PMC9924914 35943854

[44] Chu SH, Loucks EB, Kelsey KT, Gilman SE, Agha G, Eaton CB, et al. Sex-specific epigenetic mediators between early life social disadvantage and adulthood BMI. Epigenomics. 2018;10(6):707–22. 29888956 10.2217/epi-2017-0146 PMC6367732

[45] Loucks EB, Huang YT, Agha G, Chu S, Eaton CB, Gilman SE, et al. Epigenetic mediators between childhood socioeconomic disadvantage and mid-life body mass index: the New England family study. Psychosom Med. 2016;78(9):1053. 27768648 10.1097/PSY.0000000000000411 PMC7380568

[46] Wang YZ, Zhao W, Ammous F, Song Y, Du J, Shang L, et al. DNA methylation mediates the association between individual and neighborhood social disadvantage and cardiovascular risk factors. Front Cardiovasc Med. 2022;19(9):848768. 10.3389/fcvm.2022.848768 PMC9162507 35665255

[47] Mayeda ER, Glymour MM, Quesenberry CP, Whitmer RA. Inequalities in dementia incidence between six racial and ethnic groups over 14 years. Alzheimers Dement. 2016;12(3):216–24. 26874595 10.1016/j.jalz.2015.12.007 PMC4969071

[48] Barnes LL, Bennett DA. Alzheimer’s disease in African Americans: risk factors and challenges for the future. Health Aff (Millwood). 2014;33(4):580–6. 24711318 10.1377/hlthaff.2013.1353 PMC4084964

[49] Brewster P, Barnes L, Haan M, Johnson JK, Manly JJ, Nápoles AM, et al. Progress and future challenges in aging and diversity research in the United States. Alzheimers Dement. 2019;15(7):995–1003. 30240574 10.1016/j.jalz.2018.07.221 PMC7021489

[50] Rajan KB, Weuve J, Barnes LL, Wilson RS, Evans DA. Prevalence and incidence of clinically diagnosed Alzheimer’s disease dementia from 1994 to 2012 in a population study. Alzheimers Dement. 2019;15(1):1–7. 30195482 10.1016/j.jalz.2018.07.216 PMC6531287

[51] Pickle LW, Mungiole M, Gillum RF. Geographic variation in stroke mortality in Blacks and Whites in the United States. Stroke. 1997;28(8):1639–47. 9259762 10.1161/01.str.28.8.1639

[52] Liu C, Murchland AR, VanderWeele TJ, Blacker D. Eliminating racial disparities in dementia risk by equalizing education quality: A sensitivity analysis. Soc Sci Med. 2022;312:115347. 36162365 10.1016/j.socscimed.2022.115347 PMC9990698

[53] Rodriguez FS, Aranda MP, Lloyd DA, Vega WA. Racial and ethnic disparities in dementia risk among individuals with low education. Am J Geriatr Psychiatry. 2018;26(9):966–76. 30005921 10.1016/j.jagp.2018.05.011

[54] Rutter EC, Tyas SL, Maxwell CJ, Law J, O’Connell ME, Konnert CA, et al. Association between functional social support and cognitive function in middle-aged and older adults: a protocol for a systematic review. BMJ Open. 2020;10(4):e037301. 32265252 10.1136/bmjopen-2020-037301 PMC7245373

[55] Dominguez LJ, Veronese N, Vernuccio L, Catanese G, Inzerillo F, Salemi G, et al. Nutrition, physical activity, and other lifestyle factors in the prevention of cognitive decline and dementia. Nutrients. 2021;13(11):4080. 34836334 10.3390/nu13114080 PMC8624903

[56] Hsieh N, Liu H, Zhang Z. Perceived discrimination and incident dementia among older adults in the United States: the buffering role of social relationships. J Gerontol B Psychol Sci Soc Sci. 2024;79(6):gbae059. 10.1093/geronb/gbae059 PMC11125403 38587492

[57] Glymour MM, Manly JJ. Lifecourse social conditions and racial and ethnic patterns of cognitive aging. Neuropsychol Rev. 2008;18(3):223–54. 18815889 10.1007/s11065-008-9064-z

[58] Bell ML, Ebisu K. Environmental inequality in exposures to airborne particulate matter components in the United States. Environ Health Perspect. 2012;120(12):1699–704. 22889745 10.1289/ehp.1205201 PMC3546368

[59] Lee M, Whitsel E, Avery C, Hughes TM, Griswold ME, Sedaghat S, et al. Variation in population attributable fraction of dementia associated with potentially modifiable risk factors by race and ethnicity in the US. JAMA Netw Open. 2022;5(7):e2219672. 35793088 10.1001/jamanetworkopen.2022.19672 PMC9260480

[60] Daniels PR, Kardia SLR, Hanis CL, Brown CA, Hutchinson R, Boerwinkle E, et al. Familial aggregation of hypertension treatment and control in the Genetic Epidemiology Network of Arteriopathy (GENOA) study. Am J Med. 2004;116(10):676–81. 15121494 10.1016/j.amjmed.2003.12.032

[61] 1000 Genomes Project Consortium, Abecasis GR, Altshuler D, Auton A, Brooks LD, Durbin RM, et al. A map of human genome variation from population-scale sequencing. Nature. 2010;467(7319):1061–73. 10.1038/nature09534 PMC3042601 20981092

[62] Lezak P of NP and NMD, Lezak MD, Howieson AP of N and PDB, Howieson DB, Loring P of NDW, Loring DW, et al. Neuropsychological assessment. Oxford University Press; 2004. 1038 p.

[63] Smith JA, Mosley Jr TH, Turner ST, Kardia SL. Shared genetic effects among measures of cognitive function and leukoaraiosis. Edited by Amit Agrawal. 2012;39.

[64] Jaeger J. Digit Symbol Substitution Test. J Clin Psychopharmacol. 2018;38(5):513–9. 30124583 10.1097/JCP.0000000000000941 PMC6291255

[65] Davies G, Armstrong N, Bis JC, Bressler J, Chouraki V, Giddaluru S, et al. Genetic contributions to variation in general cognitive function: a meta-analysis of genome-wide association studies in the CHARGE consortium (*N*=53 949). Mol Psychiatry. 2015;20(2):183–92. 25644384 10.1038/mp.2014.188 PMC4356746

[66] Jack CR, Twomey CK, Zinsmeister AR, Sharbrough FW, Petersen RC, Cascino GD. Anterior temporal lobes and hippocampal formations: normative volumetric measurements from MR images in young adults. Radiology. 1989;172(2):549–54. 2748838 10.1148/radiology.172.2.2748838

[67] Smith JA, Turner ST, Sun YV, Fornage M, Kelly RJ, Mosley TH, et al. Complexity in the genetic architecture of leukoaraiosis in hypertensive sibships from the GENOA Study. BMC Med Genomics. 2009;7(2):16. 10.1186/1755-8794-2-16 PMC2679055 19351393

[68] Jack CR, O’Brien PC, Rettman DW, Shiung MM, Xu Y, Muthupillai R, et al. FLAIR histogram segmentation for measurement of leukoaraiosis volume. J Magn Reson Imaging. 2001;14(6):668–76. 11747022 10.1002/jmri.10011 PMC2755497

[69] Fortin JP, Fertig E, Hansen K. shinyMethyl: interactive quality control of Illumina 450k DNA methylation arrays in R. F1000Research; 2014 [cited 2021 Aug 26]. Available from: https://f1000research.com/articles/3-175 10.12688/f1000research.4680.1 PMC4176427 25285208

[70] Xu Z, Niu L, Li L, Taylor JA. ENmix: a novel background correction method for Illumina HumanMethylation450 BeadChip. Nucleic Acids Res. 2016;44(3):e20–e20. 26384415 10.1093/nar/gkv907 PMC4756845

[71] Fortin JP, Triche TJ Jr, Hansen KD. Preprocessing, normalization and integration of the Illumina HumanMethylationEPIC array with minfi. Bioinformatics. 2017;33(4):558–60. 28035024 10.1093/bioinformatics/btw691 PMC5408810

[72] Aryee MJ, Jaffe AE, Corrada-Bravo H, Ladd-Acosta C, Feinberg AP, Hansen KD, et al. Minfi: a flexible and comprehensive Bioconductor package for the analysis of Infinium DNA methylation microarrays. Bioinformatics. 2014;30(10):1363–9. 24478339 10.1093/bioinformatics/btu049 PMC4016708

[73] Niu L, Xu Z, Taylor JA. RCP: a novel probe design bias correction method for Illumina Methylation BeadChip. Bioinformatics. 2016;32(17):2659–63. 27153672 10.1093/bioinformatics/btw285 PMC5013906

[74] Lehne B, Drong AW, Loh M, Zhang W, Scott WR, Tan ST, et al. A coherent approach for analysis of the Illumina HumanMethylation450 BeadChip improves data quality and performance in epigenome-wide association studies. Genome Biol. 2015;16(1):37. 25853392 10.1186/s13059-015-0600-x PMC4365767

[75] Pidsley R, Zotenko E, Peters TJ, Lawrence MG, Risbridger GP, Molloy P, et al. Critical evaluation of the Illumina MethylationEPIC BeadChip microarray for whole-genome DNA methylation profiling. Genome Biol. 2016;17(1):208. 27717381 10.1186/s13059-016-1066-1 PMC5055731

[76] Houseman EA, Accomando WP, Koestler DC, Christensen BC, Marsit CJ, Nelson HH, et al. DNA methylation arrays as surrogate measures of cell mixture distribution. BMC Bioinformatics. 2012;13(1):86. 22568884 10.1186/1471-2105-13-86 PMC3532182

[77] Du P, Zhang X, Huang CC, Jafari N, Kibbe WA, Hou L, et al. Comparison of Beta-value and M-value methods for quantifying methylation levels by microarray analysis. BMC Bioinformatics. 2010;11(1):587. 21118553 10.1186/1471-2105-11-587 PMC3012676

[78] Weisenberger CDJ, Laird PW. Comprehensive DNA methylation analysis on the Illumina® Infinium® Assay Platform. :4.

[79] Weir DR. Quality control report for genotypic data. Project: Health and Retirement Study. [Internet]. 2021 [cited 2024 Nov 3]. Available from: https://hrs.isr.umich.edu/sites/default/files/genetic/HRS-QC-Report-Phase-4_Nov2021_FINAL.pdf

[80] Gao X, Jia M, Zhang Y, Breitling LP, Brenner H. DNA methylation changes of whole blood cells in response to active smoking exposure in adults: a systematic review of DNA methylation studies. Clin Epigenet. 2015;7(1):113. 10.1186/s13148-015-0148-3 PMC4609112 26478754

[81] Associates W&. National establishment time-series (nets) database. Walls & Associates Oakland; 2014.

[82] Desktop EA. Redlands. CA: Environmental Systems Research Institute. 2011;

[83] Silverman BW. Density estimation for statistics and data analysis. Routledge; 2018.

[84] Johnston K, Ver Hoef JM, Krivoruchko K, Lucas N. Using ArcGIS geostatistical analyst. Vol. 380. Esri Redlands; 2001.

[85] Hirsch JA, Moore KA, Clarke PJ, Rodriguez DA, Evenson KR, Brines SJ, et al. Changes in the built environment and changes in the amount of walking over time: longitudinal results from the multi-ethnic study of atherosclerosis. Am J Epidemiol. 2014;180(8):799–809. 25234431 10.1093/aje/kwu218 PMC4188343

[86] Hirsch JA, Moore KA, Barrientos-Gutierrez T, Brines SJ, Zagorski MA, Rodriguez DA, et al. Built environment change and change in BMI and waist circumference: multi-ethnic study of atherosclerosis. Obesity. 2014;22(11):2450–7. 25136965 10.1002/oby.20873 PMC4224985

[87] CDC, Census Tract Level State Maps of the Modified Retail Food Environment Index (mRFEI). Available from: https://www.cdc.gov/obesity/downloads/census-tract-level-state-maps-mrfei_TAG508.pdf

[88] Hoehner CM, Schootman M. Concordance of commercial data sources for neighborhood-effects studies. J Urban Health. 2010;87(4):713–25. 20480397 10.1007/s11524-010-9458-0 PMC2900563

[89] Hirsch JA, Grengs J, Schulz A, Adar SD, Rodriguez DA, Brines SJ, et al. How much are built environments changing, and where?: Patterns of change by neighborhood sociodemographic characteristics across seven U.S. metropolitan areas. Social Science & Medicine. 2016;169:97–105. 10.1016/j.socscimed.2016.09.032 PMC5075249 27701020

[90] Bureau UC. Census.gov. [cited 2022 Nov 7]. Summary File 1 Dataset. Available from: https://www.census.gov/data/datasets/2000/dec/summary-file-1.html

[91] Bureau UC. Census.gov. [cited 2022 Nov 7]. Summary File 3 Dataset. Available from: https://www.census.gov/data/datasets/2000/dec/summary-file-3.html

[92] Bureau UC. Census.gov. [cited 2022 Nov 7]. American Community Survey (ACS). Available from: https://www.census.gov/programs-surveys/acs

[93] Bureau UC. Census.gov. [cited 2022 Nov 7]. American Community Survey 3-Year Data (2007-2013). Available from: https://www.census.gov/data/developers/data-sets/acs-3year.html

[94] Diez Roux AV, Merkin SS, Arnett D, Chambless L, Massing M, Nieto FJ, et al. Neighborhood of residence and incidence of coronary heart disease. N Engl J Med. 2001;345(2):99–106. 11450679 10.1056/NEJM200107123450205

[95] Benjamini Y, Hochberg Y. Controlling the false discovery rate: a practical and powerful approach to multiple testing. J Roy Stat Soc: Ser B (Methodol). 1995;57(1):289–300.

[96] Du J, Zhou X, Hao W, Liu Y, Smith JA, Mukherjee B. Methods for large-scale single mediator hypothesis testing: possible choices and comparisons. arXiv; 2022 [cited 2022 Sep 17]. Available from: http://arxiv.org/abs/2203.13293 10.1002/gepi.22510 PMC10329872 36465006

[97] Cerin E. Building the evidence for an ecological model of cognitive health. Health Place. 2019;60:102206. 31797770 10.1016/j.healthplace.2019.102206

[98] Wu YT, Prina AM, Jones A, Matthews FE, Brayne C. The built environment and cognitive disorders: results from the Cognitive Function and Ageing Study II. Am J Prev Med. 2017;53(1):25–32. 28082001 10.1016/j.amepre.2016.11.020 PMC5478362

[99] Cohn-Schwartz E. Pathways From social activities to cognitive functioning: the role of physical activity and mental health. Innov Aging. 2020;4(3):igaa015. 32665981 10.1093/geroni/igaa015 PMC7325149

[100] Clarke PJ, Weuve J, Barnes L, Evans DA, Mendes de Leon CF. Cognitive decline and the neighborhood environment. Annals of Epidemiology. 2015;25(11):849–54. 10.1016/j.annepidem.2015.07.001 PMC4609590 26253697

[101] Besser LM, Rodriguez DA, McDonald N, Kukull WA, Fitzpatrick AL, Rapp SR, et al. Neighborhood built environment and cognition in non-demented older adults: the Multi-Ethnic Study of Atherosclerosis. Soc Sci Med. 2018;1(200):27–35. 10.1016/j.socscimed.2018.01.007 PMC5893410 29355828

[102] Magaziner J, Cadigan DA, Hebel JR, Parry RE. Health and living arrangements among older women: does living alone increase the risk of illness? J Gerontol. 1988;43:M127–33. 3418033 10.1093/geronj/43.5.m127

[103] Clarke PJ, Ailshire JA, House JS, Morenoff JD, King K, Melendez R, et al. Cognitive function in the community setting: the neighbourhood as a source of ‘cognitive reserve’? J Epidemiol Community Health. 2012;66(8):730–6. 21515547 10.1136/jech.2010.128116 PMC3387518

[104] Besser LM. Neighborhood built environment characteristics and cognition in non-demented older adults [Ph.D.]. [United States -- North Carolina]: The University of North Carolina at Chapel Hill; [cited 2023 Apr 15]. Available from: https://www.proquest.com/docview/1917681682/abstract/12A28C3159BD447CPQ/1

[105] Marottoli RA, de Leon CFM, Glass TA, Williams CS, Cooney LM Jr, Berkman LF. Consequences of driving cessation: decreased out-of-home activity levels. The Journals of Gerontology: Series B. 2000;55(6):S334–40. 10.1093/geronb/55.6.s334 11078110

[106] Cassarino M, Setti A. Environment as ‘Brain Training’: a review of geographical and physical environmental influences on cognitive ageing. Ageing Res Rev. 2015;1(23):167–82. 10.1016/j.arr.2015.06.003 26144974

[107] Finlay J, Esposito M, Tang S, Gomez-Lopez I, Sylvers D, Judd S, et al. Fast-food for thought: retail food environments as resources for cognitive health and wellbeing among aging Americans? Health Place. 2020;1(64):102379. 10.1016/j.healthplace.2020.102379 PMC7480653 32838895

[108] Finlay J, Esposito M, Kim MH, Gomez-Lopez I, Clarke P. Closure of ‘third places’? Exploring potential consequences for collective health and wellbeing. Health Place. 2019;1(60):102225. 10.1016/j.healthplace.2019.102225 PMC6934089 31622919

[109] Buonocore JJ, Lee HJ, Levy JI. The influence of traffic on air quality in an urban neighborhood: a community–university partnership. Am J Public Health. 2009;99(Suppl 3):S629–35. 19890168 10.2105/AJPH.2008.149138 PMC2774178

[110] Kwate NOA. Fried chicken and fresh apples: racial segregation as a fundamental cause of fast food density in black neighborhoods. Health Place. 2008;14(1):32–44. 17576089 10.1016/j.healthplace.2007.04.001

[111] Perrin AJ, Caren N, Skinner AC, Odulana A, Perrin EM. The unbuilt environment: culture moderates the built environment for physical activity. BMC Public Health. 2016;16(1):1227. 27919241 10.1186/s12889-016-3866-3 PMC5139009

[112] Eliasoph N, Lichterman P. Culture in Interaction. Am J Sociol. 2003;108(4):735–94.

[113] Rahmani A, Najand B, Sonnega A, Akhlaghipour G, Mendez MF, Assari S, et al. Intersectional effects of race and educational attainment on memory function of middle-aged and older adults with Alzheimer’s disease. J Racial and Ethnic Health Disparities. 2024;11(1):81–91. 10.1007/s40615-022-01499-w 36576695

[114] Geronimus AT. The weathering hypothesis and the health of African-American women and infants: evidence and speculations. Ethn Dis. 1992;2(3):207–21. 1467758

[115] Opdebeeck C, Martyr A, Clare L. Cognitive reserve and cognitive function in healthy older people: a meta-analysis. Aging Neuropsychol Cogn. 2016;23(1):40–60. 10.1080/13825585.2015.1041450 25929288

[116] Grasser LR, Jovanovic T. Neural impacts of stigma, racism, and discrimination. biological psychiatry: cognitive neuroscience and neuroimaging. 2022;7(12):1225–34. 10.1016/j.bpsc.2022.06.012 35811064

[117] Assari S. Understanding America: unequal economic returns of years of schooling in Whites and Blacks. World J Educ Res. 2020;7(2):78–92. 32582861 10.22158/wjer.v7n2p78 PMC7314384

[118] Assari S. Unequal gain of equal resources across racial groups. Int J Health Policy Manag. 2017;7(1):1–9. 10.15171/ijhpm.2017.90 PMC5745862 29325397

[119] Williams DR. Black-White differences in blood pressure: the role of social factors. Ethn Dis. 1992;2(2):126–41. 1467751

[120] Freese J. Genetics and the social science explanation of individual outcomes. Am J Sociol. 2008;114(S1):S1-35. 10.1086/592208 19569399

[121] Farrer LA, Cupples LA, Haines JL, Hyman B, Kukull WA, Mayeux R, et al. Effects of age, sex, and ethnicity on the association between apolipoprotein E genotype and Alzheimer disease. A meta-analysis. APOE and Alzheimer Disease Meta Analysis Consortium. JAMA. 1997;278(16):1349–56. 9343467

[122] Graff-Radford NR, Green RC, Go RCP, Hutton ML, Edeki T, Bachman D, et al. Association between apolipoprotein E genotype and Alzheimer disease in African American subjects. Arch Neurol. 2002;59(4):594–600. 11939894 10.1001/archneur.59.4.594

[123] Dai JY, Stanford JL, LeBlanc M. A multiple-testing procedure for high-dimensional mediation hypotheses. J Am Stat Assoc. 2022;117(537):198–213. 35400115 10.1080/01621459.2020.1765785 PMC8991388

[124] Huang YT. Genome-wide analyses of sparse mediation effects under composite null hypotheses. The Annals of Applied Statistics. 2019;13(1):60–84.

[125] Zeng P, Shao Z, Zhou X. Statistical methods for mediation analysis in the era of high-throughput genomics: current successes and future challenges. Comput Struct Biotechnol J. 2021;26(19):3209–24. 10.1016/j.csbj.2021.05.042 PMC8187160 34141140

[126] Blum MGB, Valeri L, Fran çois O, Cadiou S, Siroux V, Lepeule J, et al. Challenges raised by mediation analysis in a high-dimension setting. Environmental Health Perspectives. 2020;128(5):055001. 10.1289/EHP6240 PMC7263455 32379489

[127] Clark-Boucher D, Zhou X, Du J, Liu Y, Needham BL, Smith JA, et al. Methods for mediation analysis with high-dimensional DNA methylation data: possible choices and comparison [Internet]. medRxiv; 2023 [cited 2023 Nov 7]. p. 2023.02.10.23285764. Available from: https://www.medrxiv.org/content/10.1101/2023.02.10.23285764v1 10.1371/journal.pgen.1011022 PMC10655967 37934796

[128] Song Y, Zhou X, Zhang M, Zhao W, Liu Y, Kardia SLR, et al. Bayesian shrinkage estimation of high dimensional causal mediation effects in omics studies. Biometrics. 2020;76(3):700–10. 31733066 10.1111/biom.13189 PMC7228845

[129] Chén OY, Crainiceanu C, Ogburn EL, Caffo BS, Wager TD, Lindquist MA. High-dimensional multivariate mediation with application to neuroimaging data. Biostatistics. 2018;19(2):121–36. 28637279 10.1093/biostatistics/kxx027 PMC5862274

[130] Huang YT. Variance component tests of multivariate mediation effects under composite null hypotheses. Biometrics. 2019;75(4):1191–204. 31009061 10.1111/biom.13073

[131] Alzheimer’s disease facts and figures. Alzheimer’s & Dementia. 2021;17(3):327–406. 10.1002/alz.12328 33756057

[132] Tang MX, Cross P, Andrews H, Jacobs DM, Small S, Bell K, et al. Incidence of AD in African-Americans, Caribbean Hispanics, and Caucasians in northern Manhattan. Neurology. 2001;56(1):49–56. 11148235 10.1212/wnl.56.1.49

[133] Reitz C, Jun G, Naj A, Rajbhandary R. Variants in the ATP-Binding Cassette Transporter (ABCA7), Apolipoprotein E ϵ 4, and the risk of late-onset Alzheimer disease in African Americans. JAMA. 2013;309(14):1483–92. 23571587 10.1001/jama.2013.2973 PMC3667653

