# Supplementary material for: Neighborhood environment associations with cognitive function and structural brain measures in older African Americans
Source: BMC Med. 2025 Jan 13;23:15. doi: 10.1186/s12916-024-03845-7 (PMC11727707; doi:10.1186/s12916-024-03845-7)
Supplement: Supplementary file 1 — Additional File 1: Fig. S1. Flow diagram illustrating sample sizes for neighborhood density and neighborhood socioeconomic disadvantage analyses for cognitive measures in GENOA AA. Fig. S2. Flow diagram illustrating sample sizes for neighborhood density and neighborhood socioeconomic disadvantage analyses for white matter hyperintensity in GENOA AA. [file 12916_2024_3845_MOESM1_ESM.docx]

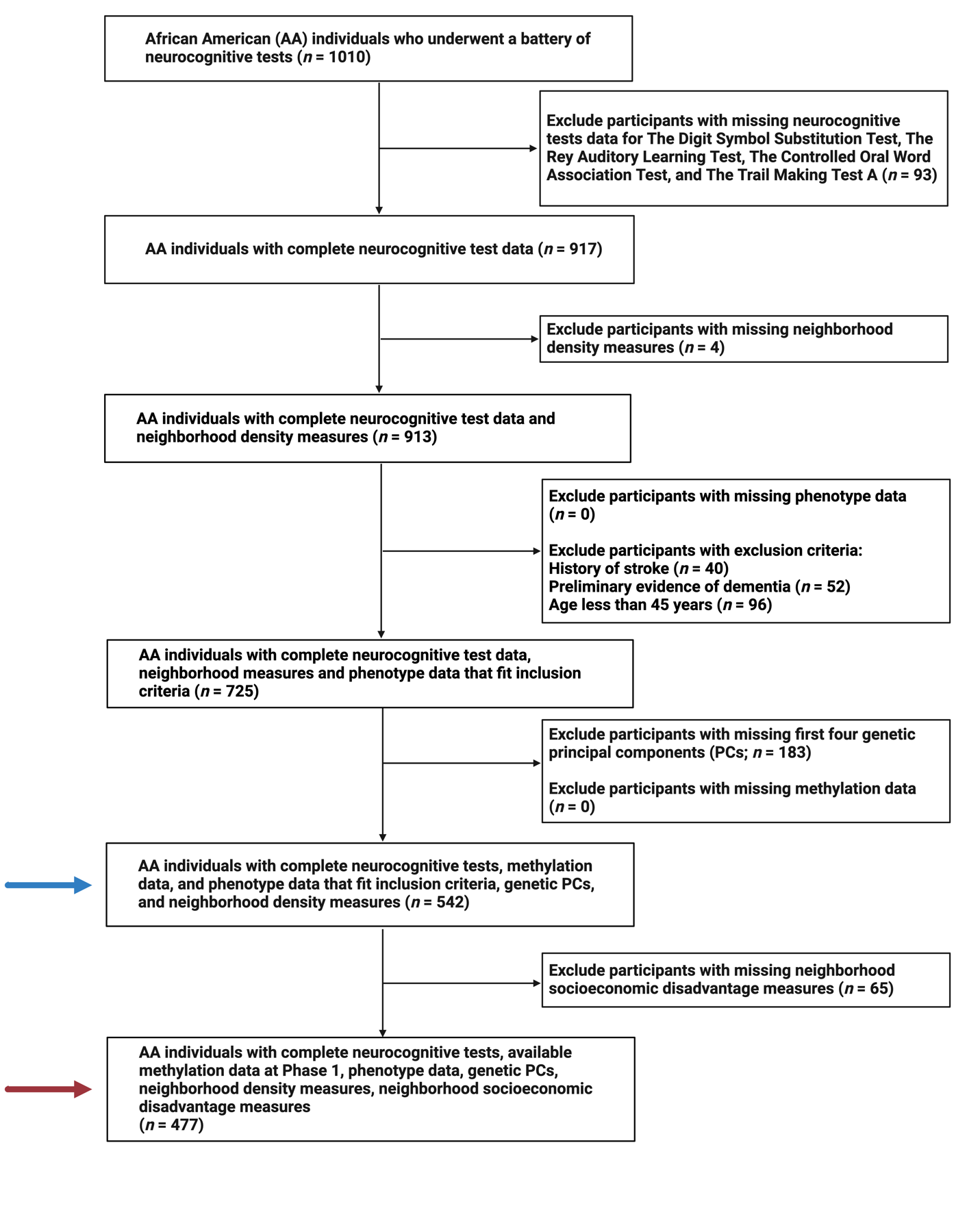


**Figure S1: Flow diagram illustrating sample sizes for neighborhood density and neighborhood socioeconomic disadvantage analyses for cognitive measures in GENOA AA.**

Flow diagram illustrating sample sizes for neighborhood density analyses (Model 1a, n=542; blue arrow) and neighborhood socioeconomic disadvantage analyses (Model 2a, n=477; red arrow) for cognitive measures in GENOA AA.


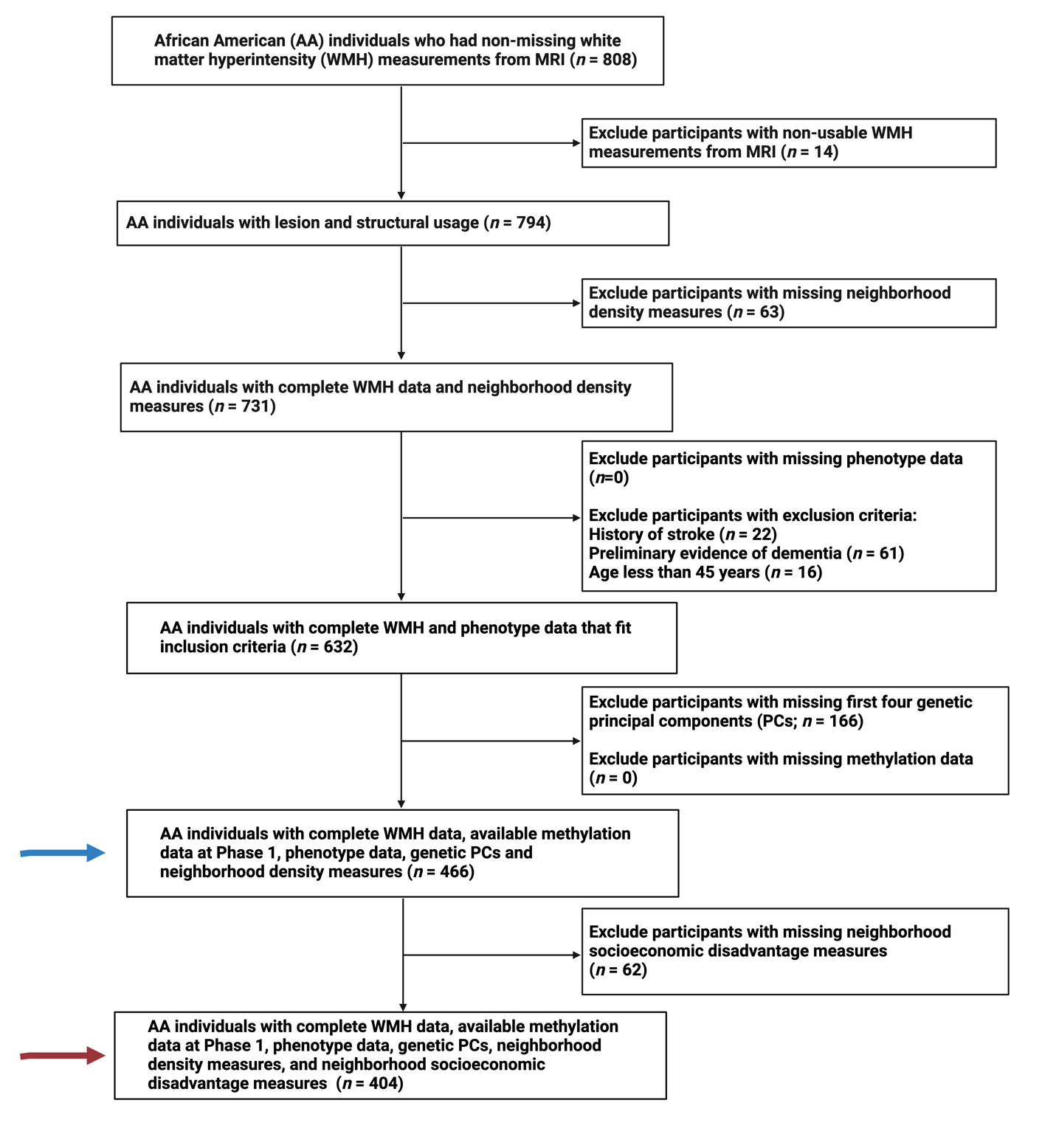


**Figure S2: Flow diagram illustrating sample sizes for neighborhood density and neighborhood socioeconomic disadvantage analyses for white matter hyperintensity in GENOA AA.** Flow diagram illustrating sample sizes for neighborhood density analyses, (Model 1b, n=466; blue arrow) and neighborhood socioeconomic disadvantage analyses (Model 2b, n=404; red arrow) for white matter hyperintensity in GENOA AA.
