# Supplementary material for: Neighborhood environment associations with cognitive function and structural brain measures in older African Americans
Source: BMC Med. 2025 Jan 13;23:15. doi: 10.1186/s12916-024-03845-7 (PMC11727707; doi:10.1186/s12916-024-03845-7)
Supplement: Supplementary file 2 — Additional File 2: Supplementary Methods [file 12916_2024_3845_MOESM2_ESM.docx]

**Supplementary Methods**

*General cognitive function*

The following four cognitive domains were evaluated a year after Phase II, on average, as part of GMBI(62,63):

1. The Weschler Adult Intelligence Scale-Revised: Digit Symbol Substitution Test (DSST) measured complex visual attention, sustained and focused concentration, response speed and visuomotor coordination. The DSST measures executive function of working memory(64). In this test, participants matched symbols to numbers according to a key located at the top of the page. The DSST score comprised the number of symbols correctly matched within 90 seconds. Scores ranged from 3 symbols to 88 symbols correctly matched within 90 seconds.
2. The Controlled Oral Word Association Test (COWA-FAS) tested for verbal fluency (phonetic association) and language. This test requires participants to name as many animals as possible that start with the letters F, A, and S in 1 minute. The score consisted of the total number of admissible animal names generated.
3. The Rey Auditory Verbal Learning Test (RAVLT) measured delayed recall, relating to the cognitive functions of new learning, immediate memory span and vulnerability to interference in learning and recognition memory. Its score was determined by the number of words recalled after a 30-minute delay. Scores ranged from 0 to 15.
4. The Trail Making Test A (TMTA) evaluated visual conceptual tracking as participants need to connect a set of 25 circles quickly and accurately. TMTA provides information on the cognitive functions of visual search, scanning, processing speed and executive functions. The natural logarithm of seconds to completion for the task was used and recoded so that higher scores indicate better cognitive function. The maximum was 240 seconds to complete.

*Neighborhood environment* *exposures*

1. GIS-based measures

Population densities of recreational, social, and healthy food environments were derived from GIS(84) data using Dun and Bradstreet data as compiled by Walls and Associates in the National Establishment Series (NETS) database(81) for 1996-2015. Addresses were geocoded using the TeleAtlas EZ-Locate web-based geocoding software (Tele Atlas North America, Inc., Lebanon, New Hampshire). NETS yearly datasets were categorized based on Standard Industrial Classification (SIC) codes. Densities per square mile were created for 0.5-,1-, and 3-mile buffers around the home addresses of GENOA participants at Phase I using ArcGIS V.9.3 (ESRI, Inc., Redlands, California)(82,83). Densities were calculated using two approaches: 1) simple densities per square mile within the buffer region and 2) kernel densities per square mile within the buffer region, with greater weighting towards resources located closer to the home of a participant. Total density scores by category were created by adding together densities from each type of establishment.

For each participant, we estimated the densities for the following destinations: fast-food restaurants (chain and non-chain), total physical activity facilities, total social engagement destinations, and alcohol outlets. Summary density measures were also created for densities of unfavorable food stores (with and without alcohol), healthy (favorable) food stores, popular walking destinations, and total food stores.

Fast food restaurants are places that specialize in low preparation time foods that are eaten cafeteria-style or take-away (SIC #581203, except for coffee shops (#58120304)). Physical activity facilities measure was created using 114 SIC codes consisting of the recreational and physical activity establishments such as indoor conditioning, dance, bowling, golf, team and racquet sports, and water activities derived from lists used in previous studies(30,31). Healthy food availability was defined using healthy food stores such as fruit and vegetable markets (SIC #5431) and supermarkets (grocery stores (SIC #5411) with at least $2 million in annual sales or at least 25 employees or name being on standardized supermarket chain name lists as described in other studies)(29). Social engagement destinations, consisting of places which promote social interaction, were derived from 430 SIC codes based on previous work(88,89). These SIC codes include locations such as beauty shops and barbers, sports entertainment, exercise facilities, amusements, libraries, museums and art galleries, religious organizations, eating and dining places. Alcohol outlets were identified as liquor stores and on-site drinking places (restaurants and nightclubs/bars).

Categories for favorable food stores consisted of supermarkets (chain and non-chain) and fruit and vegetable markets. Unfavorable food stores (without alcohol) included convenience stores, bakeries/nuts/candy/ice cream stores, and fast-food restaurants (chain and non-chain). Unfavorable food stores with alcohol included alcohol outlets. Popular walking destinations were created from six different categories including postal service, drug stores and pharmacy, banks and credit unions, food stores (non-beverage), eating and dining places (non-beverage) and drinking places (non-alcoholic). Total food stores variable was calculated from the sum of favorable food stores, neutral food stores, unfavorable food stores and other eating places.

The modified retail food environment index (MRFEI) measured the number of healthy and less healthy food retailers within census tracts across states, based on typical food offerings in specific retail stores(87). The MREI was a proportion calculated as the number of favorable food stores divided by the total of favorable and unfavorable stores (with and without alcohol outlets). The MRFEI represents the proportion of all food retailers in a given census tract that are healthy and ranges from 0 or “food desert” (e.g., no healthy food vendors) to 1 or “healthy” food vendors only. MRFEI variables were calculated for 0.5-, 1- and 3-mile buffer regions.

1. Census-based measures

Neighborhood socioeconomic disadvantage was assessed using data collected in the 2000 U.S. Census,(90,91) American Community Survey (ACS) 2005-2009(92), and ACS 2007-2011(93) estimates. Data was linked to GENOA participant data (Phase I; 1995-2000) by Census tract using Census and ACS estimates for the closest time period. A composite index was previously developed using factor analysis to determine which socioeconomic indicator variables from the Census can be meaningfully combined into a summary score. Six variables representing the dimensions of wealth and income (log of the median household income; log of the median value of housing units; and percent of household with interest, dividend or net rental income), education (the percentage of adults 25 years of age or older who had completed high school and the percentage of adults 25 years of age or older who had completed college (i.e., Bachelor’s degree)), and occupation (the percentage of employed persons 16 years of age or older in executive, managerial or professional specialty occupations) were used to characterize neighborhood socioeconomic disadvantage for each census tract(94). Z-scores for each census tract were estimated for each variable, and neighborhood socioeconomic disadvantage was defined as the sum of Z-scores from the six variables, with higher scores indicating more disadvantage.
