# Supplementary material for: Neighborhood environment associations with cognitive function and structural brain measures in older African Americans
Source: BMC Med. 2025 Jan 13;23:15. doi: 10.1186/s12916-024-03845-7 (PMC11727707; doi:10.1186/s12916-024-03845-7)
Supplement: Supplementary file 3 — Additional File 3: Table S1. Pearson’s correlations among the cognitive/WMH outcomes (N = 466). Table S2. Pearson’s correlations among neighborhood socioeconomic disadvantage and neighborhood simple density measures per square mile for 1-mile buffer size (N = 542). Table S3. Associations among neighborhood socioeconomic disadvantage and neighborhood simple density measures per square mile for 1-mile buffer size after adjusting for census tract population density (N= 542). Table S4. Pearson’s correlations among neighborhood socioeconomic disadvantage and simple and kernel densities per square mile for 1-mile buffer size. Table S5. Associations between simple density of neighborhood destinations per square mile for ½-, 1- and 3- mile buffer sizes and cognitive function/WMH. Table S6. Associations between simple density of neighborhood destinations per square mile for ½-, 1- and 3- mile buffer sizes and cognitive measures (N = 477). Table S7. Associations between kernel density of neighborhood destinations per square mile for ½-, 1- and 3- mile buffer sizes and cognitive function/WMH. Table S8. Associations between kernel density of neighborhood destinations per square mile for ½-, 1- and 3- mile buffer sizes and cognitive measures (N = 477). [file 12916_2024_3845_MOESM3_ESM.docx]

| Table S1. Pearson’s correlations among the cognitive/WMH outcomes (N=466) | | | | | | |
| --- | --- | --- | --- | --- | --- | --- |
|  | General cognitive function | RAVLT | DSST | COWA-FAS | TMTA | WMH |
| General cognitive function | 1 |  |  |  |  |  |
| RAVLT | 0.597*** | 1 |  |  |  |  |
| DSST | 0.897*** | 0.425*** | 1 |  |  |  |
| COWA-FAS | 0.670*** | 0.214** | 0.483*** | 1 |  |  |
| TMTA | 0.792*** | 0.290*** | 0.681*** | 0.304*** | 1 |  |
| WMH | -0.335*** | -0.276*** | -0.322*** | -0.119 | -0.272*** | 1 |
| Abbreviations: RAVLT: Rey Auditory Verbal Learning Test; DSST: Digit Symbol Substitution Task; COWA-FAS: Controlled Oral Word Association Test; TMTA: Trail Making Test A; WMH: White Matter Hyperintensity | | | | | | |
| * p<0.05, **p<0.01, ***p<0.001 | | | | | |  |

| Table S2. Pearson’s correlations among neighborhood socioeconomic disadvantage and neighborhood simple density measures per square mile for 1-mile buffer size (N=542) | | | | | | | | | | | | | |
| --- | --- | --- | --- | --- | --- | --- | --- | --- | --- | --- | --- | --- | --- |
| Neighborhood characteristics | Neighborhood Socioeconomic Disadvantage | Fast Food destination density | Unfavorable food stores without alcohol density | Unfavorable food stores with alcohol density | Favorable food stores density | Total physical activity destinations density | Total social engagement destinations density | Total popular walking destination density | Alcoholic drinking places density | Total food stores density | MRFEI with alcohol | MRFEI without alcohol |  |
| Neighborhood Socioeconomic Disadvantage | 1.00 |  |  |  |  |  |  |  |  |  |  |  |  |
| Fast Food destination density | -0.20** | 1.00 |  |  |  |  |  |  |  |  |  |  |  |
| Unfavorable food stores without alcohol density | 0.33*** | 0.60*** | 1.00 |  |  |  |  |  |  |  |  |  |  |
| Unfavorable food stores with alcohol density | -0.21*** | 0.93*** | 0.56*** | 1.00 |  |  |  |  |  |  |  |  |  |
| Favorable food stores density | 4.00E-3 | 0.37*** | 0.15* | 0.37*** | 1.00 |  |  |  |  |  |  |  |  |
| Total physical activity destinations density | 0.14* | 0.26*** | 0.34*** | 0.18** | 0.44*** | 1.00 |  |  |  |  |  |  |  |
| Total social engagement destinations density | 0.56*** | 0.38*** | 0.68*** | 0.34*** | 0.40*** | 0.56*** | 1.00 |  |  |  |  |  |  |
| Total popular walking destination density | 0.19** | 0.59*** | 0.52*** | 0.65*** | 0.47*** | 0.49*** | 0.72*** | 1.00 |  |  |  |  |  |
| Alcoholic drinking places density | 0.54*** | -0.05 | 0.72*** | -0.16* | -0.11 | 0.27*** | 0.52*** | 0.08 | 1.00 |  |  |  |  |
| Total food stores density | 0.17** | 0.18** | 0.13* | 0.15* | 0.89*** | 0.41*** | 0.43*** | 0.40*** | 0.05 | 1.00 |  |  |  |
| MRFEI with alcohol | 0.55*** | 0.39*** | 0.68*** | 0.37*** | 0.43*** | 0.55*** | 0.99*** | 0.77*** | 0.49*** | 0.45*** | 1.00 |  |  |
| MRFEI without alcohol | 0.45*** | 0.43*** | 0.84*** | 0.43*** | 0.18** | 0.45*** | 0.78*** | 0.76*** | 0.64*** | 0.24*** | 0.78*** | 1.00 |  |

Abbreviations: MRFEI, Modified Retail Food Environment Index

* p<0.05, **p<0.01, ***p<0.001

| Table S3. Associations among neighborhood socioeconomic disadvantage and neighborhood simple density measures per square mile for 1-mile buffer size after adjusting for census tract population density (N=542) | | | | | | | | | | | | |  |
| --- | --- | --- | --- | --- | --- | --- | --- | --- | --- | --- | --- | --- | --- |
| Neighborhood characteristics | Neighborhood Socioeconomic Disadvantage | Fast Food dest. density | Unfav. food stores without alcohol density | Unfav. food stores with alcohol density | Favorable food stores density | Total physical activity dest. density | Total social engagement dest. density | Total popular walking dest. density | Alcoholic drinking places density | Total food stores density | MRFEI with alcohol | MRFEI without alcohol | |
| Neighborhood Socioeconomic Disadvantage | 1.00 |  |  |  |  |  |  |  |  |  |  |  | |
| Fast Food dest. density | -0.02*** | 1.00 |  |  |  |  |  |  |  |  |  |  | |
| Unfav. food stores without alcohol density | 0.68*** | 0.29*** | 1.00 |  |  |  |  |  |  |  |  |  | |
| Unfav. food stores with alcohol density | 0.92*** | -0.05 | 0.68*** | 1.00 |  |  |  |  |  |  |  |  | |
| Favorable food stores density | 0.38*** | 0.14** | 0.14*** | 0.39*** | 1.00 |  |  |  |  |  |  |  | |
| Total physical activity dest. density | 0.36*** | 0.11* | 0.36*** | 0.28*** | 0.43*** | 1.00 |  |  |  |  |  |  | |
| Total social engagement dest. density | 0.52*** | 0.39*** | 0.71*** | 0.52*** | 0.40*** | 0.59*** | 1.00 |  |  |  |  |  | |
| Total popular walking dest. density | 0.68*** | 0.06 | 0.58*** | 0.75*** | 0.49*** | 0.52*** | 0.74*** | 1.00 |  |  |  |  | |
| Alcoholic drinking places density | 0.11* | 0.42*** | 0.74*** | 0.02 | -0.15** | 0.26*** | 0.51*** | 0.11* | 1.00 |  |  |  | |
| Total food stores density | 0.60*** | 0.29*** | 0.89*** | 0.61*** | 0.19*** | 0.43*** | 0.78*** | 0.77*** | 0.65*** | 1.00 |  |  | |
| MRFEI with alcohol | 0.17*** | 0.16*** | -0.12* | 0.15*** | 0.86*** | 0.33*** | 0.23*** | 0.28*** | -0.28*** | -0.03 | 1.00 |  | |
| MRFEI without alcohol | 0.16*** | 0.25*** | -0.01 | 0.12* | 0.85*** | 0.32*** | 0.28*** | 0.31*** | -0.12* | 0.09 | 0.94*** | 1.00 | |

Abbreviations: unfav., unfavorable; dest., destinations; MRFEI, Modified Retail Food Environment Index

* p<0.05, **p<0.01, ***p<0.001

Table S4. Pearson’s correlations among neighborhood socioeconomic disadvantage and simple and kernel densities per square mile for 1-mile buffer size (N=542)^a^

|  |  | Kernel density measures | | | | | | | | | | |
| --- | --- | --- | --- | --- | --- | --- | --- | --- | --- | --- | --- | --- |
|  | Neighborhood characteristics | Fast Food dest. | Unfav. food stores without alcohol | Unfav. food stores with alcohol | Favor-able food stores | Total physical activity dest. | Total social engagement dest. | Total popular walking dest. | Alcoholic drinking places | Total food stores | MRFEI with alcohol | MRFEI without alcohol |
| Simple density measures | Fast Food dest. | 0.78  *** | 0.46  *** | 0.77  *** | 0.34  *** | 0.23  *** | 0.34  *** | 0.48  *** | -0.04 | 0.14* | 0.35  *** | 0.30  *** |
|  | Unfav. food stores without alcohol) | 0.59  *** | 0.86  *** | 0.56  *** | 0.05 | 0.38  *** | 0.72  *** | 0.49  *** | 0.68  *** | -0.04 | 0.71  *** | 0.73  *** |
|  | Unfav. food stores with alcohol | 0.69  *** | 0.38  *** | 0.78  *** | 0.35  *** | 0.10 | 0.25  *** | 0.48  *** | -0.16* | 0.18  ** | 0.27  *** | 0.26  *** |
|  | Favorable food stores | 0.30  *** | 0.09 | 0.31  *** | 0.70  *** | 0.27  *** | 0.29  *** | 0.33  *** | -0.14* | 0.68  *** | 0.32  *** | 0.09 |
|  | Total physical activity dest. | 0.20  ** | 0.27  *** | 0.14* | 0.26  *** | 0.72  *** | 0.47  *** | 0.35  *** | 0.28  *** | 0.24  *** | 0.47  *** | 0.32  *** |
|  | Total social engagement dest. | 0.30  *** | 0.53  *** | 0.24  *** | 0.22  *** | 0.42  *** | 0.87  *** | 0.59  *** | 0.52  *** | 0.29  *** | 0.88  *** | 0.63  *** |
|  | Total popular walking dest. | 0.37  *** | 0.33  *** | 0.39  *** | 0.37  *** | 0.18** | 0.50  *** | 0.78  *** | 0.09 | 0.41  *** | 0.56  *** | 0.56  *** |
|  | Alcoholic drinking places | 0.14* | 0.70  *** | 0.03 | -0.22  *** | 0.38  *** | 0.65  *** | 0.18  ** | 0.93  *** | -0.19  ** | 0.62  *** | 0.66  *** |
|  | Total food stores | 0.21  *** | 0.12* | 0.16** | 0.60  *** | 0.26  *** | 0.36  *** | 0.34  *** | 0.04 | 0.74  *** | 0.38  *** | 0.21  *** |
|  | MRFEI with alcohol | 0.30  *** | 0.516  *** | 0.246  *** | 0.248  *** | 0.392  *** | 0.853  *** | 0.621  *** | 0.48  *** | 0.32  *** | 0.868  *** | 0.629  *** |
|  | MRFEI without alcohol | 0.34  *** | 0.673  *** | 0.31  *** | 0.064 | 0.292  *** | 0.688  *** | 0.660  *** | 0.634  *** | 0.128* | 0.701  *** | 0.839  *** |

a. Values with grey shading correspond to the correlations between kernel and simple densities per square mile for 1-mile buffer size

Abbreviations: unfav., unfavorable; dest., destinations; MRFEI, Modified Retail Food Environment Index

* p<0.05, **p<0.01, ***p<0.001

| Table S5. Associations between simple density of neighborhood destinations per square mile for ½-, 1- and 3- mile buffer sizes and cognitive function/WMH | | | | | | | | | | | | |
| --- | --- | --- | --- | --- | --- | --- | --- | --- | --- | --- | --- | --- |
| Neighborhood characteristics | Buffer Size | General cognitive function | | | | |  | White matter hyperintensity | | | | |
|  |  | Model 1a (N=542) | |  | Model 2a (N=477) | |  | Model 1b (N=466) | |  | Model 2b (N=404) | |
|  |  | β | P |  | β | P |  | β | P |  | β | P |
| Fast Food destination density | 1/2- mile | 5.47E-03 (-0.03, 0.04) | 0.55 |  | -0.01 (-0.05, 0.03) | 0.61 |  | 9.82E-04 (-0.03, 0.03) | 0.95 |  | -0.01 (-0.05, 0.03) | 0.75 |
|  | 1-mile | 5.05E-03 (-0.06, 0.07) | 0.53 |  | -0.03 (-0.11, 0.05) | 0.39 |  | 0.03 (-0.03, 0.09) | 0.23 |  | 0.04 (-0.02, 0.10) | 0.23 |
|  | 3-mile | 0.02 (-0.12, 0.15) | 0.70 |  | 9.56E-05 (-0.20, 0.20) | 0.99 |  | -0.10 (-0.23, 0.03) | 0.10 |  | -0.05 (-0.21, 0.11) | 0.48 |
| Unfavorable food stores (without alcohol) density | 1/2- mile | -5.77E-03 (-0.02, 0.01) | 0.17 |  | -0.01 (-0.03, 0.01) | 0.22 |  | 2.37E-03 (-0.01, 0.02) | 0.75 |  | -3.67E-04 (-0.02, 0.02) | 0.96 |
|  | 1-mile | -9.66E-03 (-0.04, 0.02) | 0.38 |  | -0.02 (-0.06, 0.02) | 0.37 |  | 0.01 (-0.02, 0.04) | 0.40 |  | 0.02 (-0.02, 0.06) | 0.24 |
|  | 3-mile | -0.03 (-0.09, 0.04) | 0.69 |  | -0.04 (-0.14, 0.06) | 0.40 |  | -0.03 (-0.09, 0.03) | 0.32 |  | -4.72E-03 (-0.09, 0.08) | 0.91 |
| Unfavorable food stores (with alcohol) density | 1/2- mile | -2.65E-03 (-0.03, 0.02) | 0.28 |  | -0.02 (-0.06, 0.02) | 0.28 |  | 0.01 (-0.02, 0.04) | 0.58 |  | -8.83E-04 (-0.02, 0.02) | 0.95 |
|  | 1-mile | -0.02 (-0.06, 0.03) | 0.26 |  | -0.05 (-0.11, 0.01) | 0.14 |  | 0.02 (-0.02, 0.06) | 0.26 |  | 0.03 (-0.01, 0.07) | 0.24 |
|  | 3-mile | -5.09E-03 (-0.09, 0.08) | 0.89 |  | -0.01 (-0.15, 0.13) | 0.86 |  | -0.05 (-0.13, 0.03) | 0.19 |  | -0.02 (-0.12, 0.08) | 0.72 |
| Favorable food stores density | 1/2- mile | 5.90E-03 (-0.09, 0.10) | 0.57 |  | 0.02 (-0.10, 0.14) | 0.69 |  | -0.01 (-0.10, 0.08) | 0.90 |  | -0.01 (-0.11, 0.09) | 0.79 |
|  | 1-mile | -0.04 (-0.21, 0.13) | 0.45 |  | -0.11 (-0.33, 0.11) | 0.31 |  | 0.02 (-0.13, 0.17) | 0.83 |  | -0.01 (-0.17, 0.15) | 0.90 |
|  | 3-mile | -0.13 (-0.52, 0.25) | 0.85 |  | -0.12 (-0.71, 0.47) | 0.68 |  | -0.13 (-0.49, 0.23) | 0.46 |  | 9.32E-04 (-0.43, 0.43) | 0.99 |
| Total physical activity destinations density | 1/2- mile | -1.16E-03 (-0.07, 0.07) | 0.59 |  | -0.01 (-0.11, 0.09) | 0.91 |  | 3.58E-03 (-0.07, 0.07) | 0.92 |  | -0.05 (-0.13, 0.03) | 0.19 |
|  | 1-mile | -0.06 (-0.20, 0.08) | 0.36 |  | -0.05 (-0.25, 0.15) | 0.58 |  | 0.03 (-0.10, 0.16) | 0.65 |  | 0.05 (-0.09, 0.19) | 0.53 |
|  | 3-mile | -0.05 (-0.32, 0.23) | 0.96 |  | -4.34E-03 (-0.41, 0.40) | 0.98 |  | -0.15 (-0.40, 0.10) | 0.23 |  | -0.03 (-0.34, 0.28) | 0.83 |
| Total social engagement destinations density | 1/2- mile | -4.91E-04 (0.00, 0.00) | 0.27 |  | -1.91E-03 (0.00, 0.00) | 0.40 |  | -4.65E-04 (0.00, 0.00) | 0.75 |  | -5.88E-04 (0.00, 0.00) | 0.74 |
|  | 1-mile | -2.19E-03 (-0.01, 0.00) | 0.29 |  | -3.59E-03 (0.00, 0.00) | 0.35 |  | 1.59E-03 (0.00, 0.01) | 0.49 |  | 3.46E-03 (0.00, 0.00) | 0.24 |
|  | 3-mile | -5.15E-03 (-0.01, 0.00) | 0.39 |  | -0.01 (-0.03, 0.01) | 0.25 |  | -1.57E-03 (-0.01, 0.01) | 0.67 |  | 1.14E-03 (0.00, 0.00) | 0.81 |
| Total popular walking destination density | 1/2- mile | 1.45E-03 (-0.01, 0.01) | 0.82 |  | 6.43E-05 (-0.02, 0.02) | 0.99 |  | 1.11E-03 (-0.01, 0.01) | 0.81 |  | -1.56E-03 (-0.02, 0.02) | 0.78 |
|  | 1-mile | -2.35E-03 (-0.02, 0.01) | 0.71 |  | -2.49E-03 (-0.02, 0.02) | 0.84 |  | 0.01 (-0.01, 0.03) | 0.38 |  | 0.01 (-0.01, 0.03) | 0.25 |
|  | 3-mile | -5.44E-03 (-0.03, 0.02) | 0.47 |  | -0.01 (-0.05, 0.03) | 0.63 |  | -0.01 (-0.03, 0.01) | 0.38 |  | -2.14E-03 (-0.02, 0.02) | 0.88 |
| Alcoholic drinking places density | 1/2- mile | -6.81E-03 (-0.05, 0.03) | 0.50 |  | -0.01 (-0.07, 0.05) | 0.65 |  | 9.17E-04 (-0.04, 0.04) | 0.96 |  | 3.21E-03 (-0.04, 0.04) | 0.88 |
|  | 1-mile | -0.01 (-0.10, 0.07) | 0.78 |  | 0.01 (-0.11, 0.13) | 0.89 |  | 1.86E-03 (-0.08, 0.08) | 0.96 |  | 0.03 (-0.07, 0.13) | 0.53 |
|  | 3-mile | -0.43 (-0.85, -0.01) | 0.12 |  | -0.71 (-1.38, -0.04) | **0.03*** |  | 0.04 (-0.36, 0.44) | 0.85 |  | 0.08 (-0.43, 0.59) | 0.74 |
| Total food stores density | 1/2- mile | -7.83E-04 (-0.01, 0.01) | 0.47 |  | -2.84E-03 (-0.02, 0.02) | 0.67 |  | -4.58E-04 (-0.01, 0.01) | 0.92 |  | -6.68E-04 (-0.02, 0.02) | 0.90 |
|  | 1-mile | -3.48E-03 (-0.02, 0.01) | 0.63 |  | -3.80E-03 (-0.02, 0.02) | 0.77 |  | 2.21E-03 (-0.01, 0.02) | 0.66 |  | 0.01 (-0.01, 0.03) | 0.38 |
|  | 3-mile | -8.58E-03 (-0.04, 0.03) | 0.82 |  | -0.02 (-0.08, 0.04) | 0.51 |  | -0.02 (-0.05, 0.01) | 0.33 |  | -3.81E-03 (-0.04, 0.04) | 0.85 |
| Modified Retail Food Environment Index (with alcohol) | 1/2- mile | 0.02 (-0.53, 0.57) | 0.54 |  | 0.10 (-0.61, 0.81) | 0.78 |  | 0.14 (-0.30, 0.58) | 0.51 |  | 0.20 (-0.27, 0.67) | 0.39 |
|  | 1-mile | 0.07 (-0.38, 0.52) | 0.73 |  | -0.13 (-0.76, 0.50) | 0.69 |  | 0.17 (-0.25, 0.59) | 0.41 |  | 0.08 (-0.37, 0.53) | 0.71 |
|  | 3-mile | -0.19 (-1.21, 0.84) | 0.66 |  | -0.17 (-1.64, 1.30) | 0.82 |  | 3.28E-04 (-0.74, 0.74) | 0.99 |  | 0.27 (-0.81, 1.35) | 0.61 |
| Modified Retail Food Environment Index (without alcohol) | 1/2- mile | 0.07 (-0.41, 0.56) | 0.25 |  | 0.26 (-0.37, 0.89) | 0.40 |  | 0.06 (-0.33, 0.45) | 0.74 |  | 0.11 (-0.32, 0.54) | 0.59 |
|  | 1-mile | 0.07 (-0.34, 0.48) | 0.93 |  | -0.05 (-0.62, 0.52) | 0.85 |  | 0.10 (-0.28, 0.48) | 0.58 |  | 0.03 (-0.40, 0.46) | 0.88 |
|  | 3-mile | -0.22 (-1.11, 0.67) | 0.88 |  | -0.01 (-1.30, 1.28) | 0.99 |  | 0.16 (-0.53, 0.85) | 0.64 |  | 0.30 (-0.66, 1.26) | 0.53 |
| Model 1a: Cognitive function = age at measurement+ PC1-4+ sex+ education+ smoking status + family (random effect) | | | | | | | | | | | | |
| Model 1b: WMH = Model 1a + total intracranial volume | | | | | | | | | | | | |
| Model 2a: Cognitive function = Model 1a + neighborhood socioeconomic disadvantage + census tract population density + family (random effect) + census tracts (random effect) | | | | | | | | | | | | |
| Model 2b: WMH = Model 2a + total intracranial volume | | | | | | | | | | | | |
| *P<0.05 | | | | | | | | | | | | |

| Table S6. Associations between simple density of neighborhood destinations per square mile for ½-, 1- and 3- mile buffer sizes and cognitive measures (N=477) | | | | | | | | | | | | | |
| --- | --- | --- | --- | --- | --- | --- | --- | --- | --- | --- | --- | --- | --- |
| Neighborhood characteristics | Buffer Size | DSST | |  | COWA-FAS | |  | RAVLT | |  | TMTA | | |
|  |  | β (95% CI) | P |  | β (95% CI) | P |  | β (95% CI) | P |  | β (95% CI) | | P |
| Fast Food destination density | 1/2- mile | -0.25 (-0.82, 0.32) | 0.37 |  | 0.01 (-0.60, 0.62) | 0.97 |  | 0.09 (-0.09, 0.27) | 0.34 |  | 0.02 (0.00, 0.04) | | 0.11 |
|  | 1-mile | -0.39 (-1.45, 0.67) | 0.45 |  | 0.27 (-0.87, 1.41) | 0.63 |  | 0.1 (-0.27, 0.47) | 0.57 |  | 0.05 (0.01, 0.09) | | **0.04*** |
|  | 3-mile | 0.17 (-2.59, 2.93) | 0.90 |  | -0.61 (-3.49, 2.27) | 0.66 |  | 0.42 (-0.52, 1.36) | 0.37 |  | 0.02 (-0.10, 0.14) | | 0.74 |
| Unfavorable food stores  (without alcohol) density | ½- mile | -0.16 (-0.43, 0.11) | 0.24 |  | -0.17 (-0.46, 0.12) | 0.25 |  | 0.05 (-0.03, 0.13) | 0.29 |  | 0.01 (-0.01, 0.03) | | 0.07 |
|  | 1-mile | -0.17 (-0.74, 0.40) | 0.55 |  | -0.19 (-0.80, 0.42) | 0.52 |  | 0.13 (-0.07, 0.33) | 0.18 |  | 0.02 (0.00, 0.04) | | 0.19 |
|  | 3-mile | -0.5 (-1.89, 0.89) | 0.47 |  | -0.4 (-1.85, 1.05) | 0.58 |  | 0.11 (-0.36, 0.58) | 0.63 |  | 0.03 (-0.03, 0.09) | | 0.32 |
| Unfavorable food stores  (with alcohol) density | ½- mile | -0.25 (-0.72, 0.22) | 0.27 |  | -0.04 (-0.53, 0.45) | 0.86 |  | -4.54E-03 | 0.95 |  | 0.02 (0.00, 0.04) | | 0.07 |
|  | 1-mile | -0.45 (-1.29, 0.39) | 0.28 |  | -0.07 (-0.97, 0.83) | 0.87 |  | 0.01 (-0.28, 0.30) | 0.94 |  | 0.04 (0.00, 0.08) | | **0.04*** |
|  | 3-mile | 0.01 (-1.79, 1.81) | 0.99 |  | -0.43 (-2.31, 1.45) | 0.64 |  | 0.27 (-0.34, 0.88) | 0.37 |  | 0.02 (-0.06, 0.10) | | 0.63 |
| Favorable food stores  density | 1/2- mile | 0.65 (-0.96, 2.26) | 0.41 |  | 0.92 (-0.82, 2.66) | 0.28 |  | -0.04 (-0.57, 0.49) | 0.89 |  | 0.02 (-0.04, 0.08) | | 0.60 |
|  | 1-mile | -1.46 (-4.36, 1.44) | 0.30 |  | 0.2 (-2.90, 3.30) | 0.89 |  | -0.31 (-1.29, 0.67) | 0.52 |  | 0.12 (-0.02, 0.26) | | 0.08 |
|  | 3-mile | -0.62 (-8.22, 6.98) | 0.87 |  | -2.59 (-10.55, 5.37) | 0.51 |  | 0.39 (-2.18, 2.96) | 0.76 |  | 0.08 (-0.29, 0.45) | | 0.66 |
| Total physical activity  destinations density | 1/2- mile | -0.49 (-1.86, 0.88) | 0.47 |  | -0.62 (-2.05, 0.81) | 0.38 |  | 0.08 (-0.37, 0.53) | 0.73 |  | -0.02 (-0.08, 0.04) | | 0.48 |
|  | 1-mile | -1.07 (-3.60, 1.46) | 0.39 |  | -1.18 (-3.88, 1.52) | 0.37 |  | 0.44 (-0.42, 1.30) | 0.30 |  | 0.05 (-0.07, 0.17) | | 0.38 |
|  | 3-mile | 0.54 (-4.77, 5.85) | 0.84 |  | -1.23 (-6.87, 4.41) | 0.66 |  | 1.28 (-0.56, 3.12) | 0.16 |  | 0.04 (-0.21, 0.29) | | 0.76 |
| Total social engagement  destinations density | 1/2- mile | -0.03 (-0.09, 0.03) | 0.24 |  | -0.04 (-0.10, 0.02) | 0.23 |  | 0.01 (-0.01, 0.03) | 0.27 |  | 1.23E-03 (0.00, 0.00) | | 0.35 |
|  | 1-mile | -0.06 (-0.16, 0.04) | 0.26 |  | -0.03 (-0.13, 0.07) | 0.61 |  | 0.02 (-0.02, 0.06) | 0.25 |  | 0.00 (0.00, 0.00) | | 0.36 |
|  | 3-mile | -0.07 (-0.23, 0.09) | 0.41 |  | -0.07 (-0.25, 0.11) | 0.42 |  | 0.01 (-0.05, 0.07) | 0.78 |  | 3.87E-03 (0.00, 0.00) | | 0.32 |
| Total popular walking  destination density | 1/2- mile | 4.97E-03 (-0.20, 0.21) | 0.96 |  | -0.09 (-0.29, 0.11) | 0.41 |  | 0.05 (-0.01, 0.11) | 0.13 |  | 3.92E-03 (0.00, 0.00) | | 0.35 |
|  | 1-mile | -0.05 (-0.38, 0.28) | 0.77 |  | 0.02 (-0.33, 0.37) | 0.88 |  | 0.09 (-0.01, 0.19) | 0.09 |  | 0.01 (-0.01, 0.03) | | 0.20 |
|  | 3-mile | -0.04 (-0.55, 0.47) | 0.88 |  | -0.14 (-0.67, 0.39) | 0.59 |  | 0.06 (-0.12, 0.24) | 0.49 |  | 0.01 (-0.01, 0.03) | | 0.50 |
| Alcoholic drinking  places density | 1/2- mile | -0.27 (-1.03, 0.49) | 0.46 |  | -0.44 (-1.26, 0.38) | 0.27 |  | 0.25 (0.00, 0.50) | **4.75E-02*** |  | 0.01 (-0.03, 0.05) | | 0.56 |
|  | 1-mile | 0.16 (-1.51, 1.83) | 0.85 |  | -0.93 (-2.67, 0.81) | 0.28 |  | 0.62 (0.05, 1.19) | **0.03*** |  | -3.11E-03 (-0.08, 0.08) | | 0.94 |
|  | 3-mile | -9.57 (-18.29, -0.85) | **0.03*** |  | -2.33 (-11.54, 6.88) | 0.61 |  | -0.8 (-3.78, 2.18) | 0.59 |  | 0.42 (0.01, 0.83) | | **0.04*** |
| Total food stores  density | 1/2- mile | -0.03 (-0.21, 0.15) | 0.74 |  | -0.14 (-0.32, 0.04) | 0.14 |  | 0.06 (0.00, 0.12) | **4.83E-02*** |  | 4.73E-03 (0.00, 0.00) | | 0.22 |
|  | 1-mile | -0.01 (-0.36, 0.34) | 0.95 |  | -0.11 (-0.48, 0.26) | 0.53 |  | 0.1 (-0.02, 0.22) | 0.07 |  | 0.01 (-0.01, 0.03) | | 0.41 |
|  | 3-mile | -0.17 (-0.91, 0.57) | 0.63 |  | -0.2 (-0.96, 0.56) | 0.60 |  | 0.07 (-0.18, 0.32) | 0.56 |  | 0.01 (-0.03, 0.05) | | 0.42 |
| Modified Retail Food  Environment Index (with alcohol) | 1/2- mile | 4.21 (-4.98, 13.40) | 0.35 |  | 5.87 (-3.67, 15.41) | 0.21 |  | -0.69 (-3.63, 2.25) | 0.63 |  | 0.14 (-0.31, 0.59) | | 0.53 |
|  | 1-mile | -3.56 (-11.55, 4.43) | 0.36 |  | 4.15 (-4.43, 12.73) | 0.32 |  | -0.64 (-3.48, 2.20) | 0.65 |  | 0.20 (-0.19, 0.59) | | 0.28 |
|  | 3-mile | 0.26 (-18.51, 19.03) | 0.98 |  | -6.93 (-25.84, 11.98) | 0.45 |  | 1.33 (-4.47, 7.13) | 0.64 |  | 0.12 (-0.72, 0.96) | | 0.77 |
| Modified Retail Food  Environment Index (without alcohol) | 1/2- mile | 5.31 (-2.68, 13.30) | 0.18 |  | 6.2 (-2.38, 14.78) | 0.14 |  | 0.07 (-2.48, 2.62) | 0.96 |  | 0.07 (-0.34, 0.48) | | 0.72 |
|  | 1-mile | -3.29 (-10.54, 3.96) | 0.36 |  | 4.43 (-3.37, 12.23) | 0.25 |  | 0.55 (-2.02, 3.12) | 0.66 |  | 0.20 (-0.13, 0.53) | | 0.21 |
|  | 3-mile | 0.2 (-16.30, 16.70) | 0.98 |  | -0.91 (-17.82, 16.00) | 0.91 |  | 0.62 (-4.65, 5.89) | 0.81 |  | 0.06 (-0.70, 0.82) | | 0.86 |
| Abbreviations: DSST, Digit Symbol Substitution Test; COWA-FAS, Controlled Oral Word Association Test; RAVLT, Rey Auditory Verbal Learning Test; TMTA, Trail Making Test A. | | | | | | | | | | | |  |  |
| Model 2a: cognitive measure = age at measurement + PC1-4 + sex + education + smoking status + neighborhood socioeconomic disadvantage + census tract population density + family (random effect) + census tracts (random effect) | | | | | | | | | | | |  |  |
| *P<0.05 | | | |  |  |  |  |  |  |  |  | |  |

| Table S7. Associations between kernel density of neighborhood destinations per square mile for ½-, 1- and 3- mile buffer sizes and cognitive function/WMH | | | | | | | | | | | | |
| --- | --- | --- | --- | --- | --- | --- | --- | --- | --- | --- | --- | --- |
| Neighborhood characteristics | Buffer Size | General cognitive function | | | | |  | White matter hyperintensity | | | | |
|  |  | Model 1a (N=542) | |  | Model 2a (N=477) | |  | Model 1b (N=466) | |  | Model 2b (N=404) | |
|  |  | β (95% CI) | P |  | β (95% CI) | P |  | β (95% CI) | P |  | β (95% CI) | P |
| Fast Food destination density | 1/2- mile | 0.01 (-0.01, 0.03) | 0.68 |  | 4.35E-03 (-0.02, 0.02) | 0.77 |  | 0.01 (-0.01, 0.03) | 0.54 |  | -0.01 (-0.03, 0.01) | 0.46 |
|  | 1-mile | -0.03 (-0.09, 0.03) | 0.31 |  | -0.04 (-0.10, 0.02) | 0.22 |  | -0.03 (-0.09, 0.03) | 0.57 |  | 0.01 (-0.03, 0.05) | 0.62 |
|  | 3-mile | -0.05 (-0.19, 0.09) | 0.44 |  | -0.10 (-0.24, 0.04) | 0.20 |  | -0.05 (-0.19, 0.09) | 0.97 |  | 0.04 (-0.08, 0.16) | 0.45 |
| Unfavorable food stores (without alcohol) density | 1/2- mile | -1.52E-03 (-0.02, 0.02) | 0.84 |  | -7.92E-04 (-0.02, 0.02) | 0.92 |  | -1.52E-03 (-0.02, 0.02) | 0.92 |  | -1.38E-03 (-0.02, 0.02) | 0.82 |
|  | 1-mile | -0.01 (-0.03, 0.01) | 0.25 |  | -0.02 (-0.04, 0.00) | 0.26 |  | -0.01 (-0.03, 0.01) | 0.57 |  | 0.01 (-0.01, 0.03) | 0.53 |
|  | 3-mile | -0.04 (-0.10, 0.02) | 0.26 |  | -0.06 (-0.14, 0.02) | 0.14 |  | -0.04 (-0.10, 0.02) | 0.92 |  | 0.02 (-0.04, 0.08) | 0.46 |
| Unfavorable food stores (with alcohol) density | 1/2- mile | -2.05E-03 (-0.02, 0.02) | 0.86 |  | -1.71E-03 (-0.02, 0.02) | 0.89 |  | -2.05E-03 (-0.02, 0.02) | 0.98 |  | -2.70E-03 (-0.02, 0.02) | 0.77 |
|  | 1-mile | -0.03 (-0.07, 0.01) | 0.22 |  | -0.03 (-0.07, 0.01) | 0.16 |  | -0.03 (-0.07, 0.01) | 0.38 |  | 0.01 (-0.03, 0.05) | 0.42 |
|  | 3-mile | -0.04 (-0.12, 0.04) | 0.36 |  | -0.07 (-0.17, 0.03) | 0.18 |  | -0.04 (-0.12, 0.04) | 0.95 |  | 0.03 (-0.05, 0.11) | 0.43 |
| Favorable food stores density | 1/2- mile | 0.02 (-0.06, 0.10) | 0.68 |  | -3.47E-03 (-0.09, 0.08) | 0.93 |  | 0.02 (-0.06, 0.10) | 0.75 |  | -0.01 (-0.07, 0.05) | 0.70 |
|  | 1-mile | -0.04 (-0.20, 0.12) | 0.58 |  | -0.10 (-0.28, 0.08) | 0.25 |  | -0.04 (-0.20, 0.12) | 0.85 |  | 0.01 (-0.11, 0.13) | 0.90 |
|  | 3-mile | -0.18 (-0.55, 0.19) | 0.32 |  | -0.31 (-0.74, 0.12) | 0.15 |  | -0.18 (-0.55, 0.19) | 0.98 |  | 0.07 (-0.26, 0.40) | 0.64 |
| Total physical activity destinations density | 1/2- mile | 0.03 (-0.03, 0.09) | 0.33 |  | 0.04 (-0.04, 0.12) | 0.28 |  | 0.03 (-0.03, 0.09) | 0.51 |  | -0.02 (-0.08, 0.04) | 0.48 |
|  | 1-mile | -0.02 (-0.14, 0.10) | 0.80 |  | -4.32E-03 (-0.17, 0.16) | 0.95 |  | -0.02 (-0.14, 0.10) | 0.96 |  | -0.04 (-0.16, 0.08) | 0.42 |
|  | 3-mile | -0.17 (-0.46, 0.12) | 0.22 |  | -0.22 (-0.57, 0.13) | 0.19 |  | -0.17 (-0.46, 0.12) | 0.76 |  | 0.06 (-0.21, 0.33) | 0.63 |
| Total social engagement destinations density | 1/2- mile | 5.47E-04 (0.00, 0.00) | 0.68 |  | 8.09E-04 (0.00, 0.00) | 0.59 |  | 5.47E-04 (0.00, 0.00) | 1.00 |  | 1.91E-05 (0.00, 0.00) | 0.99 |
|  | 1-mile | -2.53E-03 (0.00, 0.00) | 0.28 |  | -3.37E-03 (0.00, 0.00) | 0.25 |  | -2.53E-03 (0.00, 0.00) | 0.98 |  | 4.20E-04 (0.00, 0.00) | 0.85 |
|  | 3-mile | -4.79E-03 (0.00, 0.00) | 0.23 |  | -0.01 (-0.03, 0.01) | 0.10 |  | -4.79E-03 (0.00, 0.00) | 0.97 |  | 2.09E-03 (0.00, 0.00) | 0.58 |
| Total popular walking destination density | 1/2- mile | 1.30E-03 (0.00, 0.00) | 0.78 |  | 2.24E-04 (-0.02, 0.02) | 0.97 |  | 1.30E-03 (0.00, 0.00) | 0.65 |  | -3.44E-03 (0.00, 0.00) | 0.42 |
|  | 1-mile | -3.50E-03 (-0.02, 0.02) | 0.66 |  | -0.01 (-0.03, 0.01) | 0.56 |  | -3.50E-03 (-0.02, 0.02) | 0.71 |  | 2.75E-03 (-0.02, 0.02) | 0.71 |
|  | 3-mile | -0.01 (-0.03, 0.01) | 0.38 |  | -0.02 (-0.06, 0.02) | 0.18 |  | -0.01 (-0.03, 0.01) | 0.84 |  | 0.01 (-0.01, 0.03) | 0.54 |
| Alcoholic drinking places density | 1/2- mile | 0.01 (-0.03, 0.05) | 0.76 |  | 0.01 (-0.03, 0.05) | 0.70 |  | 0.01 (-0.03, 0.05) | 0.93 |  | -0.01 (-0.05, 0.03) | 0.74 |
|  | 1-mile | -0.02 (-0.10, 0.06) | 0.68 |  | -0.01 (-0.09, 0.07) | 0.82 |  | -0.02 (-0.10, 0.06) | 0.78 |  | -0.01 (-0.07, 0.05) | 0.87 |
|  | 3-mile | -0.12 (-0.39, 0.15) | 0.33 |  | -0.14 (-0.49, 0.21) | 0.43 |  | -0.12 (-0.39, 0.15) | 0.80 |  | -0.01 (-0.28, 0.26) | 0.95 |
| Total food stores density | 1/2- mile | 1.35E-04 (0.00, 0.00) | 0.98 |  | 1.04E-03 (-0.02, 0.02) | 0.84 |  | 1.35E-04 (0.00, 0.00) | 0.61 |  | -2.57E-03 (0.00, 0.00) | 0.51 |
|  | 1-mile | -0.01 (-0.03, 0.01) | 0.48 |  | -0.01 (-0.03, 0.01) | 0.51 |  | -0.01 (-0.03, 0.01) | 0.98 |  | 2.36E-03 (-0.02, 0.02) | 0.74 |
|  | 3-mile | -0.02 (-0.06, 0.02) | 0.34 |  | -0.03 (-0.07, 0.01) | 0.20 |  | -0.02 (-0.06, 0.02) | 0.71 |  | 0.01 (-0.03, 0.05) | 0.62 |
| Modified Retail Food Environment Index (with alcohol) | 1/2- mile | 0.34 (-0.25, 0.93) | 0.25 |  | 0.03 (-0.64, 0.70) | 0.92 |  | 0.34 (-0.25, 0.93) | 0.38 |  | 0.28 (-0.15, 0.71) | 0.18 |
|  | 1-mile | -0.03 (-0.54, 0.48) | 0.92 |  | -0.14 (-0.67, 0.39) | 0.59 |  | -0.03 (-0.54, 0.48) | 0.32 |  | 0.20 (-0.19, 0.59) | 0.29 |
|  | 3-mile | -0.31 (-1.15, 0.53) | 0.45 |  | -0.70 (-1.78, 0.38) | 0.19 |  | -0.31 (-1.15, 0.53) | 0.69 |  | 0.23 (-0.57, 1.03) | 0.56 |
| Modified Retail Food Environment Index (without alcohol) | 1/2- mile | 0.51 (-0.02, 1.04) | 0.05 |  | 0.24 (-0.37, 0.85) | 0.43 |  | 0.51 (-0.02, 1.04) | 0.40 |  | 0.27 (-0.14, 0.68) | 0.18 |
|  | 1-mile | 0.04 (-0.43, 0.51) | 0.85 |  | -0.08 (-0.59, 0.43) | 0.75 |  | 0.04 (-0.43, 0.51) | 0.59 |  | 0.12 (-0.25, 0.49) | 0.50 |
|  | 3-mile | -0.20 (-0.96, 0.56) | 0.60 |  | -0.42 (-1.36, 0.52) | 0.37 |  | -0.20 (-0.96, 0.56) | 0.49 |  | 0.21 (-0.50, 0.92) | 0.54 |
| Abbreviations: CI, confidence interval  Model 1a: Cognitive function = age at measurement+ PC1-4+ sex+ education+ smoking status + family (random effect) | | | | | | | | | | | | |
| Model 1b: WMH = Model 1a + total intracranial volume | | | | | | | | | | | | |
| Model 2a: Cognitive function = Model 1a + neighborhood socioeconomic disadvantage + census tract population density + family (random effect) + census tracts (random effect) | | | | | | | | | | | | |
| Model 2b: WMH = Model 2a + total intracranial volume | | | | | | | | | | | | |
| *P<0.05 | | | | | | | | | | | |  |

| Table S8. Associations between kernel density of neighborhood destinations per square mile for ½-, 1- and 3- mile buffer sizes and cognitive measures (N=477) | | | | | | | | | | | | |
| --- | --- | --- | --- | --- | --- | --- | --- | --- | --- | --- | --- | --- |
| Neighborhood characteristics | Buffer Size | DSST | |  | COWA-FAS | |  | RAVLT | |  | TMTA | |
|  |  | β (95% CI) | P |  | β (95% CI) | P |  | β (95% CI) | P |  | β (95% CI) | P |
| Fast Food destination density | 1/2- mile | -0.13 (-0.50, 0.24) | 0.49 |  | 0.20 (-0.21, 0.61) | 0.33 |  | 0.11 (-0.01, 0.23) | 0.08 |  | 0.01 (-0.01, 0.03) | 0.14 |
|  | 1-mile | -0.49 (-1.27, 0.29) | 0.20 |  | 0.04 (-0.80, 0.88) | 0.93 |  | 0.08 (-0.19, 0.35) | 0.52 |  | 0.05 (0.01, 0.09) | **0.01*** |
|  | 3-mile | -1.16 (-3.12, 0.80) | 0.23 |  | -0.10 (-2.22, 2.02) | 0.92 |  | -0.04 (-0.73, 0.65) | 0.90 |  | 0.08 (-0.02, 0.18) | 0.09 |
| Unfavorable food stores (without alcohol) density | 1/2- mile | -0.07 (-0.27, 0.13) | 0.50 |  | 0.05 (-0.17, 0.27) | 0.67 |  | 0.07 (0.01, 0.13) | 0.05 |  | 0.01 (0.01, 0.01) | 0.13 |
|  | 1-mile | -0.15 (-0.54, 0.24) | 0.44 |  | -0.15 (-0.56, 0.26) | 0.48 |  | 0.08 (-0.04, 0.20) | 0.19 |  | 0.02 (0.00, 0.04) | **0.03*** |
|  | 3-mile | -0.71 (-1.81, 0.39) | 0.19 |  | -0.27 (-1.43, 0.89) | 0.64 |  | 0.06 (-0.33, 0.45) | 0.74 |  | 0.04 (-0.02, 0.10) | 0.11 |
| Unfavorable food stores (with alcohol) density | 1/2- mile | -0.15 (-0.46, 0.16) | 0.32 |  | 0.16 (-0.17, 0.49) | 0.33 |  | 0.05 (-0.05, 0.15) | 0.34 |  | 0.01 (-0.01, 0.03) | 0.14 |
|  | 1-mile | -0.35 (-0.96, 0.26) | 0.24 |  | -0.03 (-0.68, 0.62) | 0.92 |  | 0.03 (-0.17, 0.23) | 0.78 |  | 0.04 (0.02, 0.06) | **0.01*** |
|  | 3-mile | -0.80 (-2.17, 0.57) | 0.23 |  | -0.26 (-1.73, 1.21) | 0.72 |  | 0.02 (-0.45, 0.49) | 0.92 |  | 0.05 (-0.01, 0.11) | 0.12 |
| Favorable food stores density | 1/2- mile | 0.13 (-0.95, 1.21) | 0.80 |  | 0.29 (-0.89, 1.47) | 0.62 |  | -0.07 (-0.44, 0.30) | 0.70 |  | 0.02 (-0.02, 0.06) | 0.47 |
|  | 1-mile | -0.76 (-3.01, 1.49) | 0.49 |  | -0.06 (-2.51, 2.39) | 0.96 |  | -0.43 (-1.19, 0.33) | 0.25 |  | 0.08 (-0.02, 0.18) | 0.11 |
|  | 3-mile | -2.68 (-8.17, 2.81) | 0.32 |  | -1.35 (-7.27, 4.57) | 0.64 |  | -1.05 (-2.91, 0.81) | 0.26 |  | 0.14 (-0.13, 0.41) | 0.30 |
| Total physical activity destinations density | 1/2- mile | -0.09 (-1.05, 0.87) | 0.85 |  | 0.24 (-0.82, 1.30) | 0.65 |  | 0.24 (-0.09, 0.57) | 0.13 |  | -0.01 (-0.05, 0.03) | 0.51 |
|  | 1-mile | -0.63 (-2.59, 1.33) | 0.52 |  | -0.40 (-2.52, 1.72) | 0.70 |  | 0.39 (-0.28, 1.06) | 0.23 |  | 0.01 (-0.09, 0.11) | 0.89 |
|  | 3-mile | -3.30 (-7.71, 1.11) | 0.13 |  | -2.59 (-7.35, 2.17) | 0.27 |  | 0.76 (-0.77, 2.29) | 0.31 |  | 0.15 (-0.05, 0.35) | 0.15 |
| Total social engagement destinations density | 1/2- mile | -0.01 (-0.05, 0.03) | 0.54 |  | 0.01 (-0.03, 0.05) | 0.68 |  | 0.02 (0.00, 0.04) | **0.01*** |  | 9.26E-04 (0.00, 0.00) | 0.29 |
|  | 1-mile | -0.05 (-0.13, 0.03) | 0.23 |  | -0.04 (-0.12, 0.04) | 0.32 |  | 0.02 (0.00, 0.04) | 0.12 |  | 3.24E-03 (0.00, 0.00) | 0.07 |
|  | 3-mile | -0.09 (-0.23, 0.05) | 0.16 |  | -0.07 (-0.21, 0.07) | 0.31 |  | 1.44E-03 (-0.04, 0.04) | 0.95 |  | 4.38E-03 (0.00, 0.00) | 0.18 |
| Total popular walking destination density | 1/2- mile | -0.03 (-0.17, 0.11) | 0.68 |  | 0.01 (-0.15, 0.17) | 0.91 |  | 0.04 (0.00, 0.08) | 0.07 |  | 0.01 (0.01, 0.01) | **0.04*** |
|  | 1-mile | -0.03 (-0.30, 0.24) | 0.78 |  | -0.08 (-0.37, 0.21) | 0.55 |  | 0.07 (-0.01, 0.15) | 0.08 |  | 0.01 (-0.01, 0.03) | **0.03*** |
|  | 3-mile | -0.21 (-0.64, 0.22) | 0.31 |  | -0.21 (-0.66, 0.24) | 0.35 |  | 0.03 (-0.11, 0.17) | 0.69 |  | 0.01 (-0.01, 0.03) | 0.17 |
| Alcoholic drinking places density | 1/2- mile | -1.03E-04 (-0.53, 0.53) | 1.00 |  | 0.05 (-0.52, 0.62) | 0.85 |  | 0.24 (0.06, 0.42) | **0.01*** |  | 0.01 (-0.01, 0.03) | 0.46 |
|  | 1-mile | -0.07 (-1.19, 1.05) | 0.90 |  | -0.56 (-1.76, 0.64) | 0.34 |  | 0.37 (-0.02, 0.76) | 0.06 |  | 0.01 (-0.05, 0.07) | 0.61 |
|  | 3-mile | -1.55 (-6.02, 2.92) | 0.48 |  | -1.36 (-6.16, 3.44) | 0.56 |  | 0.28 (-1.33, 1.89) | 0.73 |  | 0.06 (-0.19, 0.31) | 0.60 |
| Total food stores density | 1/2- mile | -0.01 (-0.13, 0.11) | 0.85 |  | 0.01 (-0.13, 0.15) | 0.94 |  | 0.05 (0.01, 0.09) | **0.03*** |  | 4.61E-03 (0.00, 0.00) | 0.10 |
|  | 1-mile | -0.02 (-0.27, 0.23) | 0.86 |  | -0.13 (-0.40, 0.14) | 0.31 |  | 0.08 (0.00, 0.16) | 0.05 |  | 0.01 (-0.01, 0.03) | **0.04*** |
|  | 3-mile | -0.28 (-0.87, 0.31) | 0.33 |  | -0.23 (-0.86, 0.40) | 0.45 |  | 0.05 (-0.15, 0.25) | 0.60 |  | 0.02 (0.00, 0.04) | 0.16 |
| Modified Retail Food Environment Index (with alcohol) | 1/2- mile | 2.51 (-5.88, 10.90) | 0.54 |  | 4.54 (-4.24, 13.32) | 0.29 |  | -1.08 (-3.82, 1.66) | 0.42 |  | 0.04 (-0.37, 0.45) | 0.83 |
|  | 1-mile | -3.75 (-10.45, 2.95) | 0.25 |  | 3.10 (-4.25, 10.45) | 0.39 |  | -0.97 (-3.36, 1.42) | 0.41 |  | 0.08 (-0.25, 0.41) | 0.61 |
|  | 3-mile | -6.17 (-20.04, 7.70) | 0.36 |  | -7.32 (-21.82, 7.18) | 0.30 |  | -1.40 (-5.95, 3.15) | 0.53 |  | 0.27 (-0.38, 0.92) | 0.40 |
| Modified Retail Food Environment Index (without alcohol) | 1/2- mile | 4.86 (-2.76, 12.48) | 0.19 |  | 6.06 (-2.27, 14.39) | 0.14 |  | -0.59 (-3.08, 1.90) | 0.63 |  | -0.02 (-0.41, 0.37) | 0.91 |
|  | 1-mile | -3.20 (-9.43, 3.03) | 0.30 |  | 3.77 (-3.07, 10.61) | 0.26 |  | -0.31 (-2.54, 1.92) | 0.77 |  | 0.09 (-0.22, 0.40) | 0.54 |
|  | 3-mile | -3.41 (-15.54, 8.72) | 0.57 |  | -3.16 (-15.99, 9.67) | 0.61 |  | -0.99 (-5.07, 3.09) | 0.62 |  | 0.18 (-0.41, 0.77) | 0.53 |
| Abbreviations: DSST, Digit Symbol Substitution Test; COWA-FAS, Controlled Oral Word Association Test; RAVLT, Rey Auditory Verbal Learning Test; TMTA, Trail Making Test A; CI, confidence interval | | | | | | | | | | | | |
| Model 2a: cognitive measure = age at measurement + PC1-4 + sex + education + smoking status + neighborhood socioeconomic disadvantage + census tract population density + family (random effect) + census tracts (random effect) | | | | | | | | | | | | |
| *P<0.05 |  |  |  |  |  |  |  |  |  |  |  |  |
